# Generation of locus coeruleus norepinephrine neurons from human pluripotent stem cells

**DOI:** 10.1038/s41587-023-01977-4

**Published:** 2023-11-16

**Authors:** Yunlong Tao, Xueyan Li, Qiping Dong, Linghai Kong, Andrew J. Petersen, Yuanwei Yan, Ke Xu, Seth Zima, Yanru Li, Danielle K. Schmidt, Melvin Ayala, Sakthikumar Mathivanan, Andre M. M. Sousa, Qiang Chang, Su-Chun Zhang

**Affiliations:** 1 Waisman Center, University of Wisconsin-Madison, Madison, WI USA; 2 State Key Laboratory of Pharmaceutical Biotechnology, School of Life Sciences, Chemistry and Biomedicine Innovation Center (ChemBIC), Nanjing University, Nanjing, China; 3 Department of Neuroscience, Department of Neurology, University of Wisconsin-Madison, Madison, WI USA; 4 Program in Neuroscience and Behavioral Disorders, Duke-NUS Medical School, Singapore, Singapore

**Keywords:** Neural stem cells, Differentiation, Neuronal development, Cell fate and cell lineage

## Abstract

Central norepinephrine (NE) neurons, located mainly in the locus coeruleus (LC), are implicated in diverse psychiatric and neurodegenerative diseases and are an emerging target for drug discovery. To facilitate their study, we developed a method to generate 40–60% human LC-NE neurons from human pluripotent stem cells. The approach depends on our identification of ACTIVIN A in regulating LC-NE transcription factors in dorsal rhombomere 1 (r1) progenitors. In vitro generated human LC-NE neurons display extensive axonal arborization; release and uptake NE; and exhibit pacemaker activity, calcium oscillation and chemoreceptor activity in response to CO_2_. Single-nucleus RNA sequencing (snRNA-seq) analysis at multiple timepoints confirmed NE cell identity and revealed the differentiation trajectory from hindbrain progenitors to NE neurons via an *ASCL1*-expressing precursor stage. LC-NE neurons engineered with an NE sensor reliably reported extracellular levels of NE. The availability of functional human LC-NE neurons enables investigation of their roles in psychiatric and neurodegenerative diseases and provides a tool for therapeutics development.

## Abstract (web summary)

Norepinephrine neurons, a drug target for neurologic diseases, are produced from stem cells.

## Main

Norepinephrine (NE), also known as noradrenaline (NA), is mainly produced by NE neurons in the locus coeruleus (LC) of the central nervous system (CNS). There are 10,000–30,000 LC-NE neurons in non-human primates and 20,000–50,000 in humans^1,2^ projecting to nearly the entire brain^3^ through their elaborate axonal branches. The NE system is involved in arousal, wakefulness, memory, focus and attention and ‘fight or flight’ reaction^4^. Dysregulation of the NE system is linked to many neurological disorders, such as Alzheimer’s disease (AD), Parkinson’s disease (PD), congenital central hypoventilation syndrome (CCHS), sleep disorders, attention-deficit/hyperactivity disorder (ADHD), anxiety and depression^4–8^.

Central NE neurons are heterogenous, having different developmental origins, anatomical locations, connectivity and function^9,10^. LC-NE neurons, the main NE nucleus in the CNS, originate from the dorsal hindbrain rhombomere 1 (r1)^9–11^, whereas other NE nuclei develop from r2–r5 (ref. ^9^). LC-NE neurons often degenerate at an early stage of many neurodegenerative diseases^4,8^, although it is not clear why they are vulnerable and how their degeneration contributes to disease pathogenesis. Hence, the LC-NE system is an emerging target for revealing pathogenesis of, and developing therapeutics for, many neurological disorders^8,12–15^.

Studies on the LC-NE system are hindered by the lack of readily available authentic NE neurons, especially of human origin. Immortalized ‘NE-like’ cell lines (for example, PC12 and SH-SY5Y) express some related gene profiles and produce the NE neurotransmitter but do not recapitulate developmental processes and often contain mutations^16^, limiting their utility. Forced expression of NE-related transcription factors Phox2b or Phox2a generates neurons with NE phenotypes from mouse embryonic stem cells (mESCs) but is less effective in inducing expression of the NE neuronal markers tyrosine hydroxylase (TH) and dopamine β hydroxylase (DBH) in cells derived from human pluripotent stem cells (hPSCs)^17^. It is not known whether hPSC-derived or mESC-derived cells possess NE neuron functionality. BMP7 plays a positive role in mouse NE neuron generation but has a negative effect in human cells^17^, suggesting potential species difference in NE fate determination. It is currently unclear how human NE neurons, especially LC-NE neurons, are specified. Consequently, generation of functional human LC-NE neurons has not been achieved.

In this Article, we found that bone morphogenic proteins (BMPs) and transforming growth factor-beta (TGFβ), known to induce NE neuron development in the rodent brain and from mESCs^17^, do not have an obvious effect on the generation of NE neurons from hPSCs. Notably, we established that ACTIVIN A, another member of the TGFβ superfamily, is important in regulating neurogenesis and the NE fate of the hPSC-specified r1 progenitors. This finding enabled generation of 40–60% functional LC-NE neurons that resemble their in vivo counterparts from one embryonic stem cell (ESC) line (H9) and two induced pluripotent stem cell (iPSC) lines (W24B and W24M).

## Specification of dorsal hindbrain r1 progenitors from hPSCs

**Fig. 1:**
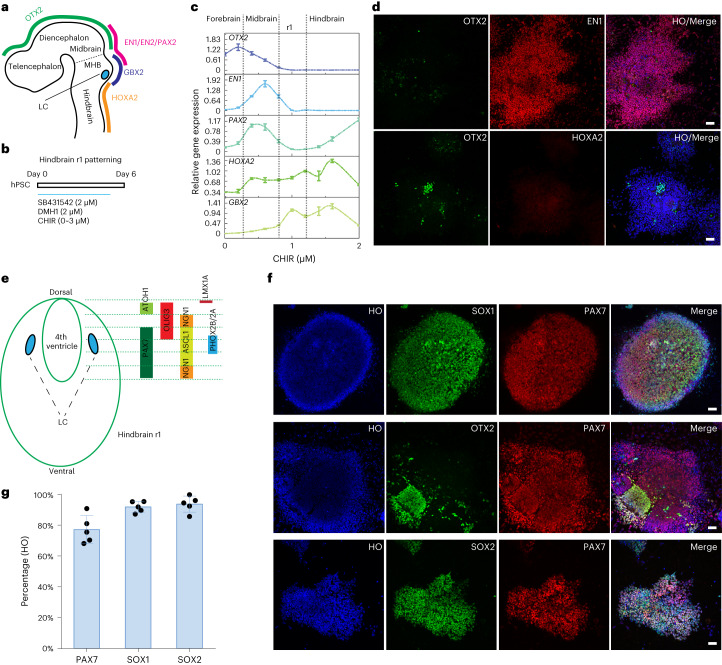
Specification of dorsal hindbrain (r1) neuroepithelia. **a**, Schematic representation of LC location along the embryonic forebrain, midbrain and hindbrain and their corresponding homeodomain transcription factors. **b**, Experimental design to pattern hindbrain r1 region from hPSCs during the first 6 d of neural induction. **c**, Expression of forebrain, midbrain and hindbrain genes under a series of CHIR99021 (CHIR) concentrations. Data are shown as mean ± s.e.m. *n* = 3 biologically independent samples for each condition. **d**, Immunostaining for OTX2, EN1 and HOXA2 in day 6 cells when treated with 1.0 µM CHIR99021 in the presence of SB431542 and DMH1. Scale bar, 50 µm. **e**, Schematic representation of the hindbrain r1 domains along the dorsal to ventral subdomains and their corresponding transcription factors. **f**, Immunostaining for PAX7, SOX1 and SOX2 at day 6 from cells treated with 1.0 µM CHIR99021 in the presence of SB431542 and DMH1. HO, Hoechst. Scale bar, 50 µm. **g**, Quantification of SOX2-, SOX1- and PAX7-expressing cells at day 6 when treated with 1.0 µM CHIR99021. Data are shown as mean ± s.d. *n* = 5 biologically independent samples for each condition. Source data

**Extended Data Fig. 1:**
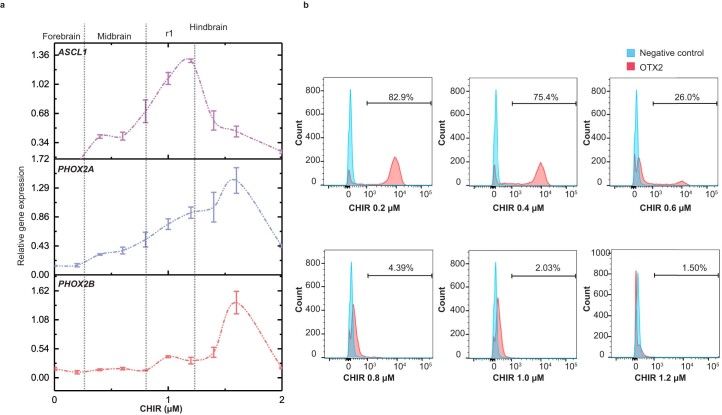
Hindbrain r1 patterning. **a**, qPCR of norepinephrine neural progenitor markers ASCL1, PHOX2A and PHOX2B under the treatment of a series of CHIR concentrations. Data are shown as mean ± SEM. n = 3 biologically independent samples for each condition. **b**, Flow cytometry quantification of OTX2 positive cell population in differentiating cells under a series of CHIR99021 (CHIR) concentrations. Source data

LC-NE neurons originate from progenitors in the dorsal hindbrain r1 during embryonic development^9^. Progenitors in the r1 segment express EN1/2 and GBX2 but not OTX2 (forebrain and midbrain marker) or HOXA2 (hindbrain marker from r2)^18^ (Fig. 1a). We first differentiated human embryonic stem cells (hESCs, H9 line) to neuroepithelial cells in the presence of BMP receptor inhibitor DMH1 (2 µM) and TGFβ receptor inhibitor SB431542 (2 µM)^19^. With an increasing concentration of WNT agonist CHIR99012 for 6 d during neuroepithelial specification (Fig. 1b), the differentiating cells downregulated the expression of *OTX2* at the mRNA level (Fig. 1c). Concurrently, midbrain markers (*EN1*, *EN2* and *PAX2*) were upregulated. At CHIR99021 concentrations above 0.6 µM, these midbrain transcription factors began to downregulate at the mRNA level, whereas the hindbrain markers (*HOXA2* and *GBX2*) were upregulated (Fig. 1c). The NE-related transcription factors *ASCL1* (also known as *MASH1*), *PHOX2A* and *PHOX2B* increased in response to increasing CHIR99021 doses (Extended Data Fig. 1a). This pattern of gene expression was confirmed by immunocytochemistry, showing expression of EN1 in the majority of the cells, whereas few were positive for OTX2 or HOXA2 at 1.0 µM (Fig. 1d). Flow cytometry also revealed the shift of the OTX2-expressing cell population in response to increasing concentrations of CHIR99021 (Extended Data Fig. 1b). Thus, we selected 1.0 µM CHIR99021 to pattern the hindbrain r1 identity from hPSCs, which matches a previous study using 1.4 µM CHIR99021 to pattern the hindbrain r2–r3 region from hPSCs^20^.

LC-NE neurons are located bilaterally at the 4th ventricle; most of their progenitors were generated from the PAX7^+^ lineage in mice, and a small population (9%) was generated from the PAX7^−^ cells in mice^10^ (Fig. 1e). Indeed, over 80% of the neuroepithelial cells at day 6, marked by SOX1 and SOX2, were PAX7 positive (Fig. 1f,g). Thus, the differentiated cells possess the dorsal r1 identity.

## Specification of NE fate from r1 progenitors by ACTIVIN A

**Fig. 2:**
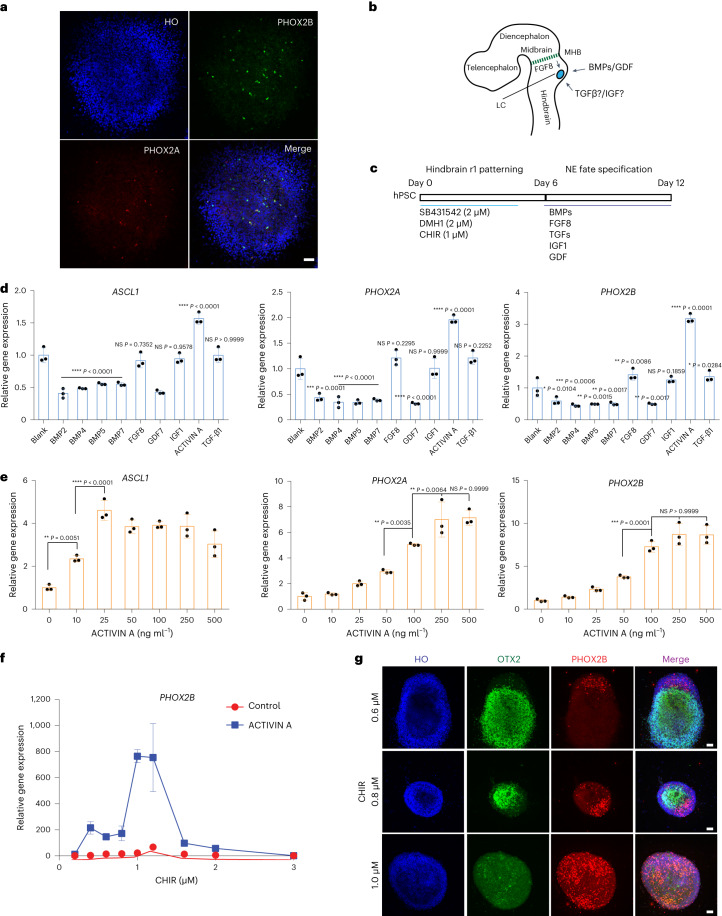
Specification of NE progenitors. **a**, Immunostaining for NE neural progenitor markers PHOX2A and PHOX2B at day 6 in cells treated with 1.0 µM CHIR99021 (CHIR). HO, Hoechst. Scale bar, 50 µm. **b**, Schematic representation of the potential morphogens that may affect NE progenitor fate specification. **c**, Experimental design to identify factors that positively affect NE progenitor specification. **d**, qPCR of NE progenitor markers *ASCL1*, *PHOX2A* and *PHOX2B* under the treatment of BMPs, FGF8, GDF7, IGF1, ACTIVIN A and TGFβ1. Data are shown as mean ± s.d. *n* = 3 biologically independent samples for each condition. The significance (versus ‘Blank’ condition) was assessed by one-way ANOVA (Dunnett’s multiple comparisons test). *P < 0.05, **P < 0.01,***P < 0.001 and ****P < 0.0001. NS, not significant. **e**, qPCR of NE progenitor markers *ASCL1*, *PHOX2A* and *PHOX2B* under a series of ACTIVIN A concentrations. Data are shown as mean ± s.d. *n* = 3 biologically independent samples for each condition. The significance was assessed by one-way ANOVA (Dunnett’s multiple comparisons test). *P < 0.05, **P < 0.01 and ***P < 0.001. NS, not significant. **f**, Relative *PHOX2B* expression in the presence or absence of ACTIVIN A (2nd week), whereas the cells were treated with a series of CHIR99021 concentrations at the first week. Data are shown as mean ± s.d. *n* = 3 biologically independent samples for each condition. **g**, Immunostaining for the regional marker OTX2 and NE progenitor marker PHOX2B at day 12 when cells were treated with 125 ng ml^−1^ ACTIVIN A. Scale bar, 50 µm. Source data

The development of NE neurons in LC depends on the activation of Ascl1, Phox2a and Phox2b in a stage-dependent manner^21–23^. Ascl1 is expressed in the progenitors, whereas Phox2a and Phox2b are expressed in post-mitotic precursors^21^. Immunocytochemical analysis of the dorsal r1 progenitors indicated that very few cells (<1%) were positive for ASCL1, PHOX2A and PHOX2B (Fig. 2a and Supplementary Fig. 1a), suggesting that additional signals are required for activating NE markers. In zebrafish, FGF8 and BMPs are essential for NE differentiation^24^ (Fig. 2b). Thus, we tested the effect of FGF8 and BMPs after hindbrain r1 patterning (Fig. 2c). Treatment of the r1 neuroepithelial cells from day 6 to day 12 with 10 ng ml^−1^ BMP2, BMP4, BMP5 and BMP7; 100 ng ml^−1^ FGF8; or 10 ng ml^−1^ GDF7 had no obvious positive effects on the expression of *ASCL1*, *PHOX2A* and *PHOX2B* (Fig. 2d). To the contrary, BMPs and GDF7 decreased the expression of *ASCL1*, *PHOX2A* and *PHOX2B*. We also observed an upregulation of the dorsal region markers (*OLIG3* and *ATOH1*) by BMPs (Supplementary Fig. 1b), suggesting that the inhibitory role of BMPs is possibly due to their dorsalization effect on the neural progenitors. Examining additional ligands in the TGFβ family, we found that ACTIVIN A (10 ng ml^−1^), but not TGFβ1 (10 ng ml^−1^), had a positive effect on the expression of *ASCL1*, *PHOX2A* and *PHOX2B* (Fig. 2d), suggesting a special role of ACTIVIN A in the specification of NE neuron progenitors from hPSCs.

We then examined the expression the NE progenitor marker *ASCL1* and NE precursor markers *PHOX2A* and *PHOX2B* in response to increasing doses of ACTIVIN A. *ASCL1* reached the maximum expression level at 25 ng ml^−1^ ACTIVIN A, and it did not change with higher concentrations of ACTIVIN A. In contrast, *PHOX2A* and *PHOX2B* reached their maximum expression level at 250 ng ml^−1^ ACTIVIN A (Fig. 2e). This pattern of ACTIVIN A effects on the expression of *ASCL1* versus *PHOX2A*/*2B* suggests that a lower dose of ACTIVIN A endows the r1 progenitors with neurogenic potential, whereas a higher concentration of ACTIVIN A specifies the ASCL1^+^ progenitor cells to *PHOX2A*-expressing or *PHOX2B*-expressing NE precursors.

**Extended Data Fig. 2:**
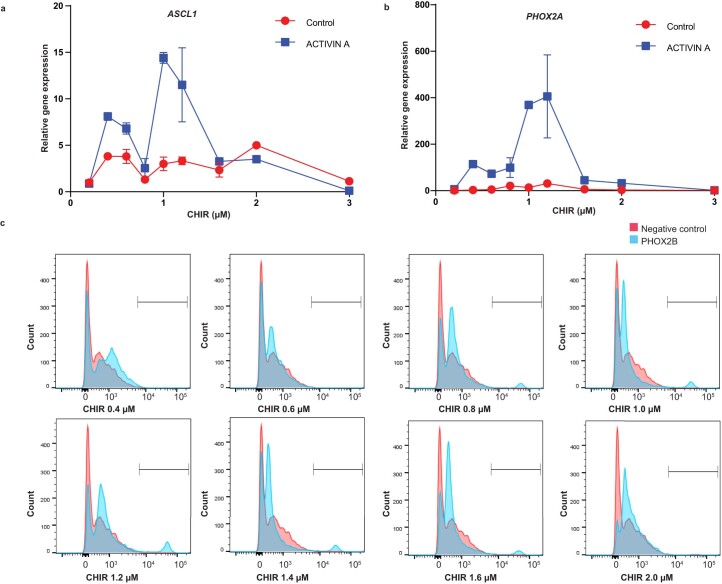
Regional dependent effect of ACTIVIN A. **a, b**, qPCR of ASCL1 and PHOX2A expression under ACTIVIN A treatment (2nd week) following a series of CHIR99021(CHIR) concentrations at the first week. Data are shown as mean ± s.e.m. n = 3 biologically independent samples for each condition. **c**, Flow cytometry quantification of PHOX2B positive cell population from cells treated with ACTIVIN A after being pre-treated with a series of CHIR concentrations. Source data

To determine whether the effect of ACTIVIN A on NE progenitor specification is specific to r1 progenitors, we first generated neuroepithelial cells with identities ranging from anterior forebrain to posterior hindbrain by using different doses of CHIR99021 and then treated them with 125 ng ml^−1^ ACTIVIN A for 6 d. We found that only cells with the rostral hindbrain identity—that is, treated with CHIR99021 from 1.0 µM to 1.4 µM—exhibited the highest level of *PHOX2B* expression as well as *ASCL1* and *PHOX2A* (Fig. 2f and Extended Data Fig. 2a,b). Immunostaining showed that PHOX2B^+^ cells were negative for OTX2 (Fig. 2g), indicating that the NE precursors can be specified only in OTX2^−^ cells. This was also confirmed by flow cytometry analysis showing the highest population of PHOX2B cells when the cultures were patterned by CHIR99021 at 1.0 µM to 1.4 µM and treated with ACTIVIN A (Extended Data Fig. 2c). Together, these results suggest that ACTIVIN A acts specifically on the dorsal r1 progenitors to generate the NE precursors.

## The temporal effect of ACTIVIN A in specifying NE precursors

**Fig. 3:**
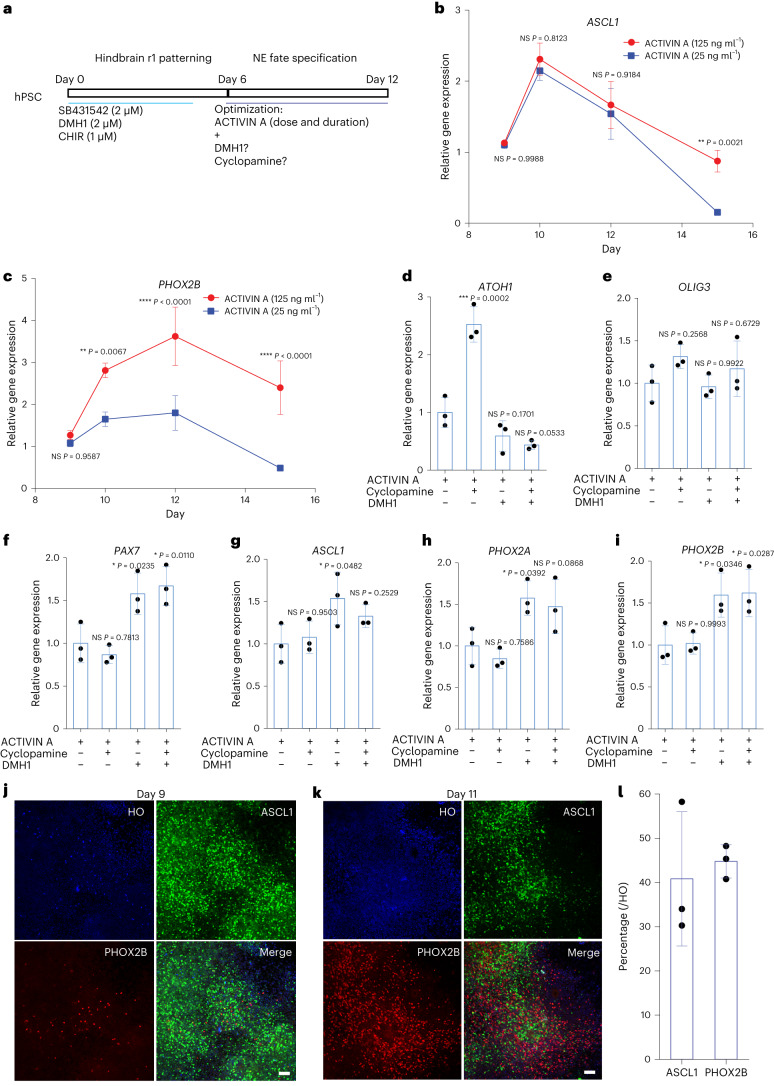
Temporal and concentration effects of ACTIVIN A on the expression of ASCL1 and PHOX2A/2B. **a**, Experimental design to optimize NE progenitor fate specification at the second stage of differentiation. CHIR, CHIR99021. **b**,**c**, qPCR of *ASCL1* and *PHOX2B* expression under 25 ng ml^−1^ and 125 ng ml^−1^ ACTIVIN A from day 9 to day 15. Data are shown as mean ± s.d. *n* = 3 biologically independent samples for each condition. The significance (comparison between 25 ng ml^−1^ and 125 ng ml^−1^ at the same timepoint) was assessed by two-way ANOVA (Sidak’s multiple comparisons test). *P < 0.05, **P < 0.01, ***P < 0.001 and ****P < 0.0001. NS, not significant. **d**–**f**, Expression of dorsal markers *ATOH1*, *OLIG3* and *PAX7* under the treatment of ACTIVIN A with or without DMH1/cyclopamine. Data are shown as mean ± s.d. *n* = 3 biologically independent samples for each condition. The significance (versus the first condition) was assessed by one-way ANOVA (Dunnett’s multiple comparisons test). *P < 0.05, **P < 0.01, ***P < 0.001 and ****P < 0.0001. **g**–**i**, Expression of NE progenitor marker genes *ASCL1*, *PHOX2A* and *PHOX2B* under the treatment of ACTIVIN A with or without DMH1/cyclopamine. Data are shown as mean ± s.d. *n* = 3 biologically independent samples for each condition. The significance (versus the first condition) was assessed by one-way ANOVA (Dunnett’s multiple comparisons test). *P < 0.05 and **P < 0.01. **j**–**l**, Immunostaining of NE progenitor markers ASCL1 and PHOX2B at day 9 (**j**) and day 11 (**k**) during differentiation under the opimized condition and their quantification. Scale bar in **j**,**k**, 50 µm. **l**, HO, Hoechst. Data are shown as mean ± s.d. *n* = 3 biologically independent samples for each condition. Source data

The differential dose dependent effect of ACTIVIN A on *ASCL1* and *PHOX2A*/*2B* expression suggests a need for sequential activation of *ASCL1* and *PHOX2A*/*2B* in NE neuron specification. Therefore, we treated the hindbrain r1 neuroepithelia at day 6 with a low dose (25 ng ml^−1^) or a high dose (125 ng ml^−1^) of ACTIVIN A and examined the expression of ASCL1and PHOX2B from day 9 to day 15 (Fig. 3a). There was no obvious difference in *ASCL1* expression between the low and high doses from day 9 to day 12 (Fig. 3b). PHOX2B expression was similar between the high-dose and low-dose groups at day 9 but higher in the high-dose condition after day 9 and peaked at day 12. Its expression level in the high-dose group was twice as that in the low-dose condition (Fig. 3c). These results indicate that *ASCL1* is activated by a low dose of ACTIVIN A at an early stage (day 6 to day 9), whereas PHOX2B is activated by a high dose at a later stage (day 9 to day 12).

As described above, many BMP family ligands negatively regulate the generation of NE precursors from hPSCs (Fig. 2d and Supplementary Fig. 1b) as opposed to mouse cells^17^. We asked whether blockade of the BMP pathway or inhibition of the Sonic hedgehog (SHH) pathway (to prevent ventralization or enhance dorsalization) facilitates NE precursor specification induced by ACTIVIN A. Treatment with SHH antagonist cyclopamine (2 µM) increased the expression of dorsal markers *ATOH1* (Fig. 3d) and *OLIG3* (does not reach significance; Fig. 3e) but with minor effects on *PAX7* (Fig. 3f), *ASCL1* (Fig. 3g) and *PHOX2A*/*2B* (Fig. 3h,i), suggesting that dorsalization at this stage has little effect on the NE precursors. In contrast, treatment with BMP antagonist DMH1 (2 µM) significantly upregulated markers of NE neural progenitors (Fig. 3f–i) without affecting *ATOH1* and *OLIG3* significantly (Fig. 3d,e), suggesting that the inhibitory role of BMPs on NE precursor fate is unlikely related to their dorsalization effect. This is further shown by the fact that the addition of cyclopamine, which induced dorsal markers, did not antagonize the effect of BMP inhibition (Fig. 3f–i). Thus, BMPs inhibit the NE precursor fate of human r1 progenitors, and BMP inhibition further improves the effect of ACTIVIN A in NE precursor specification.

With the identification of the region-dependent and time (stage)-dependent effect of ACTIVIN A as well as its coordination with the BMP antagonist DMH1, we optimized our protocol by treating the r1 progenitors with 25 ng ml^−1^ ACTIVIN A at days 6–8 and 125 ng ml^−1^ ACTIVIN A at days 9–11 in the presence of 2 µM DMH1 (Supplementary Fig. 2a). Under this condition, 92% of the cells were positive for ASCL1 at day 9, whereas 40% of the total cells were positive for ASCL1 and 45% for PHOX2B at day 11 (Fig. 3j–l).

## Characteristics of differentiated NE neurons

**Fig. 4:**
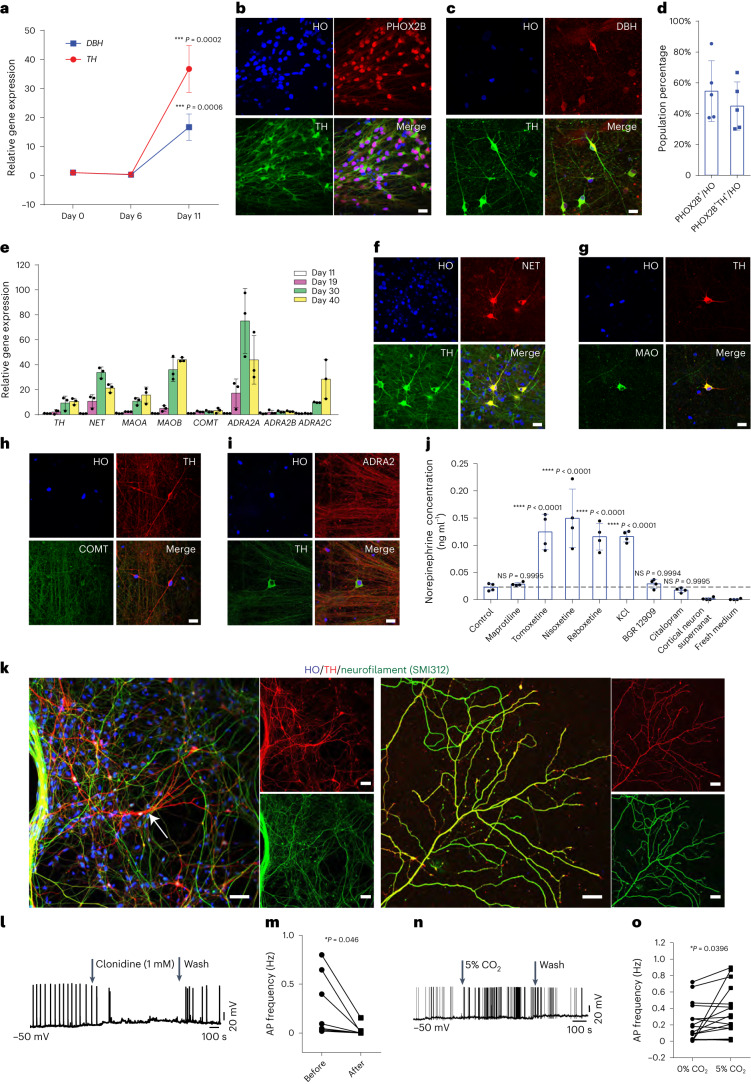
Maturation of NE neurons. **a**, qPCR of NE neuronal marker genes *TH* and *DBH* during differenitation. Data are shown as mean ± s.d. *n* = 3 biologically independent samples for each condition. The significance (versus day 0) was assessed by one-way ANOVA (Dunnett’s multiple comparisons test) for each gene. *P < 0.05, **P < 0.01, ***P < 0.001 and ****P < 0.0001. NS, not significant. **b**,**c**, Immunostaining of NE neuronal markers PHOX2B, TH and DBH at day 18 (**b**) and day 30 (**c**). HO, Hoechst. Scale bar, 20 µm. **d**, Quantification of PHOX2B and PHOX2B/TH^+^ cells in culture. Data are shown as mean ± s.d. *n* = 5 biologically independent samples for each condition. **e**, qPCR analysis of gene expression after neuronal differentiation at days 11, 19, 30 and 40. Data are shown as mean ± s.d. *n* = 3 biologically independent samples for each condition. **f**–**i**, Immunostaining of NE markers NET, MAO, COMT and ADRA2 in H9-dervied NE neurons at day 30. Scale bar, 20 µm. **j**, Supernatant NE content at week 4 under the treatment of NRIs or KCl. Data are shown as mean ± s.d. *n* = 4 biologically independent samples for each condition. Significance (versus control group) was assessed by one-way ANOVA (Dunnett’s multiple comparisons test). *P < 0.05, **P < 0.01, ***P < 0.001 and ****P < 0.0001. **k**, Immunostaining of neurofilament in NE neuron cell body, dendrites and axons. The white arrow points to the NE cell body, which is not stained by neurofilament (SM312). Scale bar, 50 µm. **l**, Representative trace of spontaneous firing before, at and after clonidine (1 mM) treatment. **m**, Quantification of the firing rate change in **l**. Data are shown as symbols and lines in the ‘before–after’ pattern. *n* = 6 neurons. **n**, Representative trace of spontaneous firing before, at and after perfusion with 5% CO_2_. **o**, Quantification of the firing rate change in **n**. Data are shown as symbols and lines in the ‘before–after’ pattern. *n* = 16 neurons. Significance was assessed by paired *t*-test (two-tailed) in **m**,**o**. Source data

**Extended Data Fig. 3:**
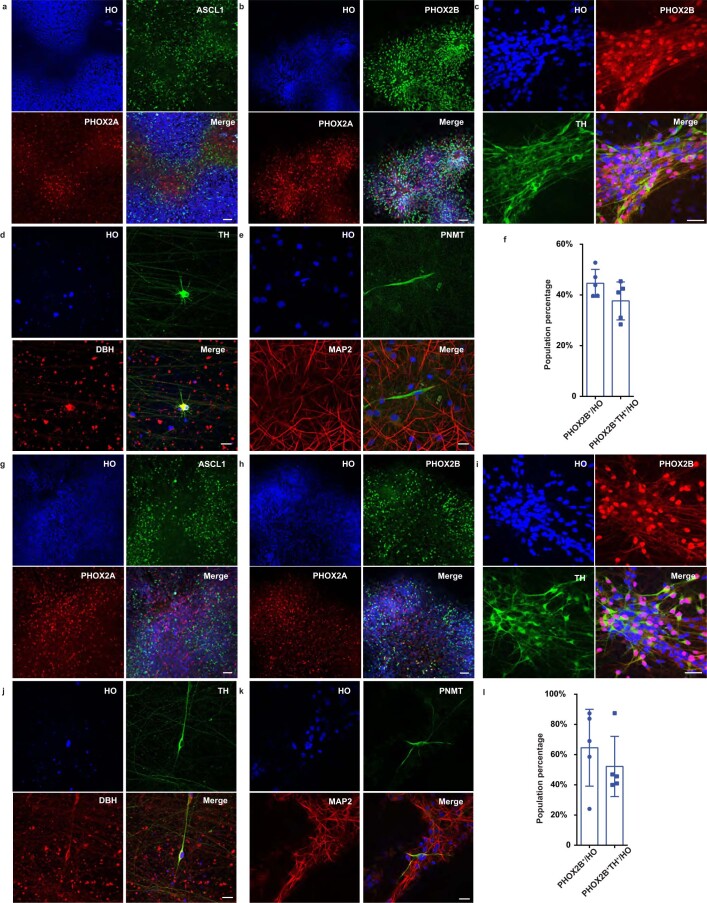
Generation of NE neurons from hiPSCs. **a–l**, Replication of the NE differentiation in W24B (a-f) and W24M (g-l) hiPSCs. a,b,g,h, Immunostaining for NE progenitor markers ASCL1, PHOX2B and PHOX2A at day 11. c,d,i,j, Immunostaining for NE markers PHOX2B, TH and DBH at day 18 (c, i) and day 30 (d,j). e,k, Immunostaining for PNMT in culture. HO, Hoechst. scale bars (a, b, g, h), 50 μm; scale bars (c-e,i-k): 20 μm. f,l, Quantification of PHOX2B and PHOX2B/TH positive cells in culture. Data are shown as mean ± s.d. n = 5 biologically independent samples for each condition. Source data

The hPSC-specified neural progenitor clusters were dissociated into single cells at days 10–12 and cultured under the neuronal differentiation condition (Supplementary Fig. 2a,b). qPCR analysis indicated that the expression of *TH* and *DBH*, the rate-limiting enzymes for synthesizing NE, increased by 16-fold and 36-fold from day 6 to day 11, respectively (Fig. 4a). The proportion of PHOX2B and TH double-positive cells was about 45% in the whole culture, whereas about 55% of the total cells were PHOX2B positive (Fig. 4b,d). By 30 d of differentiation, we observed that TH^+^ cells also co-express DBH by immunochemistry (Fig. 4c). The generation of NE progenitors and neurons was reproduced in additional two iPSC lines (line W24B and line W24M) in a similar efficiency (Extended Data Fig. 3a–d,f,h–k,m) with 40–60% of cells positive for PHOX2B at day 11 and 35–45% of cells positive for TH and PHOX2B at day 18 (Extended Data Fig. 3f,m). The iPSC-derived TH^+^ neurons co-expressed DBH by day 30 (Extended Data Fig. 3d,k).

**Extended Data Fig. 4:**
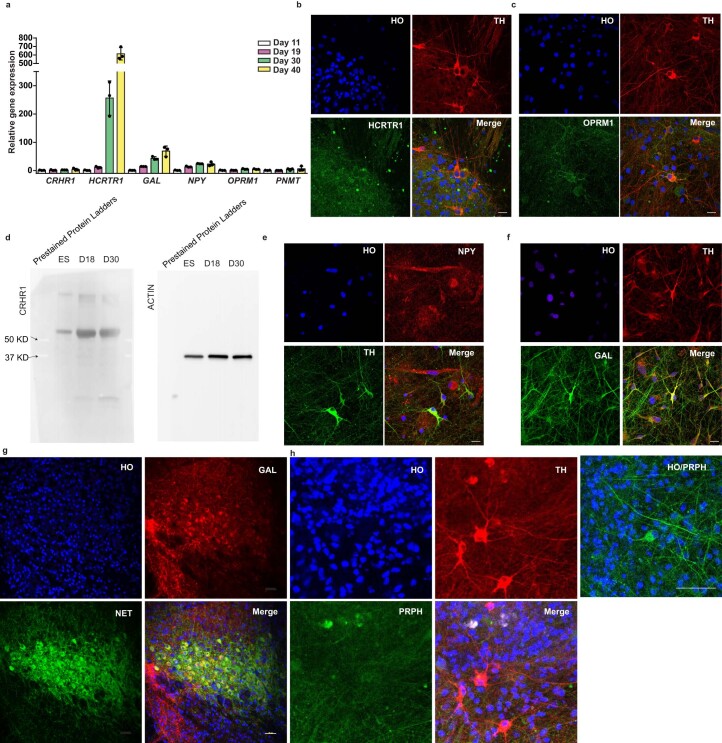
Characterization of hPSC derived NE neuron. **a**, qPCR of gene expression following NE neuronal differentiation at day 11, 19, 30 and 40. Data are shown as mean ± s.d. n = 3 biologically independent samples for each condition. **b, c**, Immunostaining for HCRT1 and OPRM1 in H9 derived NE neurons at day 30. Scale bars, 20 μm. **d**, The expression of CRHR1 by western blot at Day 0 (ES), Day 18 (D18) and Day 30 (D30) along NE differentiation. **e, f**, Immunostaining for NPY and GAL in H9 derived NE neurons at day 30. scale bars, 50 μm. **g**, Immunostaining for GAL and NET in mouse LC to demonstrate the specificity of the GAL antibody. Scale bar, 50 μm. **h**,Immunostaining for PRPH (Peripherin) in hPSC derived LC-NE neurons at day 30. A positive control to demonstrate the specificity of the peripherin antibody is provided using hPSC derived neurons. Scale bars, 20 μm. Source data

Other NE neuronal markers, such as norepinephrine transporter (*NET*), monoamine oxidase (*MAO*), catechol-*O*-methyltransferase (*COMT)* and adrenoceptor alpha 2A (*ADRA2)*, were upregulated from day 11 to day 40 (Fig. 4e). Their protein expression was confirmed by immunocytochemistry in the NE neurons at day 30 (Fig. 4f–i). NET, MAO and COMT were expressed in TH^+^, but not TH^−^, neurons at a ratio of 70%, 92% and 85%, respectively (Fig. 4f–i). LC-NE neuron activity can be regulated by corticotropin-releasing factor (CRF), hypocretin (orexin) and opioid peptide (enkephalin) through their receptors. Indeed, the gene expression of CRF receptor (CRHR1), orexin receptor (HCRTR1) and mu-opioid receptor (OPRM1) was detected in the cultures by qPCR (Extended Data Fig. 4a) and in some of the NE neurons by immunocytochemistry or western blot (Extended Data Fig. 4b–d). Subsets of LC-NE neurons co-express other peptides, such as neuropeptide Y (NPY) and galanin (GAL)^25^. Indeed, *NPY* and *GAL* mRNAs were upregulated along NE differentiation (Extended Data Fig. 4a), and their peptides were detected in some of the TH^+^ cells (Extended Data Fig. 4e,f). About 75% and 30% of TH^+^ cells co-expressed GAL and NPY, respectively, which is similar to LC neurons in rats^26^. GAL and NPY were also expressed in TH^−^ cells at the ratio of ~15% and ~20% (Extended Data Fig. 6e–g), consistent with their wide distribution in the nearby brainstem regions^27–30^. Because LC-NE neurons share many of the NE neuron markers with peripheral sympathetic NE neurons, we examined the expression of peripherin, a marker for neurons in the peripheral nervous system. As expected, the hPSC-derived NE neurons expressed TH but not peripherin (Extended Data Fig. 4h), further confirming the identity of central NE neurons.

**Extended Data Fig. 5:**
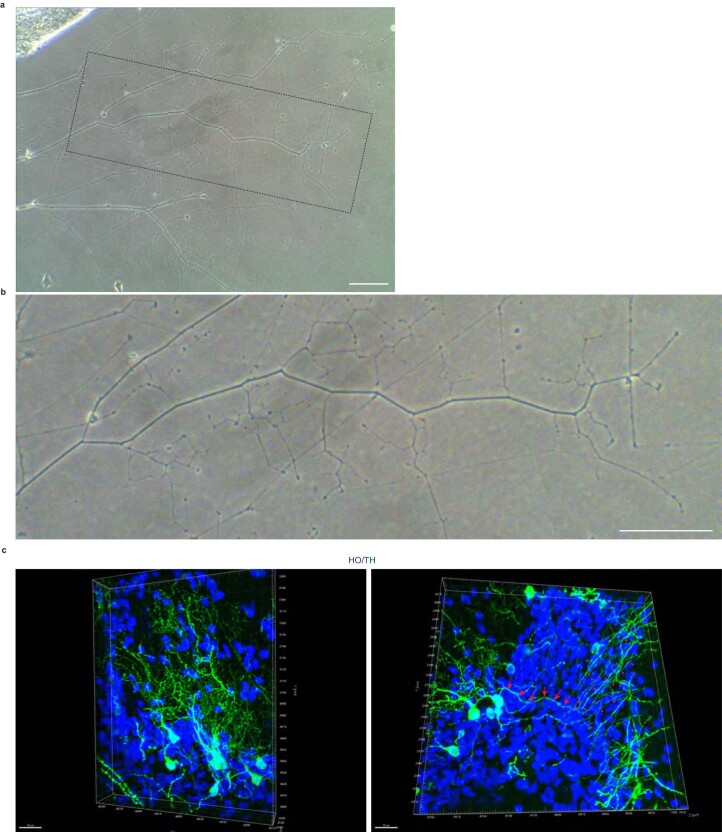
NE neuron maturation in vitro and vivo. **a, b**, Phase contrast image showing neurite (axon) branching in NE neuron cultures at 2 months. Scale bars, 0.1 mm. **c**, The 3D reconstruction view showing the dendrites and axon branches from TH+ NE neurons in the transplanted mouse cortex. The arrows point to an axon. Each arrowhead indicates an axonal branch site. Scale bars, 20 μm.

LC-NE neurons rely on massive axonal arbors to innervate large brain areas^31^. With long-term differentiation (2 months), NE neurons developed extensive axonal branches. The identity of the NE axons was confirmed by their co-labeling with TH and SMI312, an antibody that specifically labels axons (Fig. 4k and Extended Data Fig. 5a,b). As an additional demonstration, we transplanted the day 12 NE progenitors into the cerebral cortex of adult SCID mice and found that the differentiated (TH^+^) NE neurons projected fine axons with numerous branches 3 months after transplantation (Extended Data Fig. 5c). Together, these features suggest that hPSC-derived NE neurons resemble their in vivo counterparts.

Besides NE neurons, we found that some of the neurons in our cultures were positive for GABA (~5%) and CaMKII or VGLUT1 (~40%) at day 30. Few cells were positive for phenylethanolamine *N*-methyl transferase (PNMT) (<1%), whereas no 5-HT neurons were detected (Supplementary Fig. 3a–d). The PNMT^+^ cells were also observed in iPSC-derived NE cultures (Extended Data Fig. 3e,k).

**Extended Data Fig. 6:**
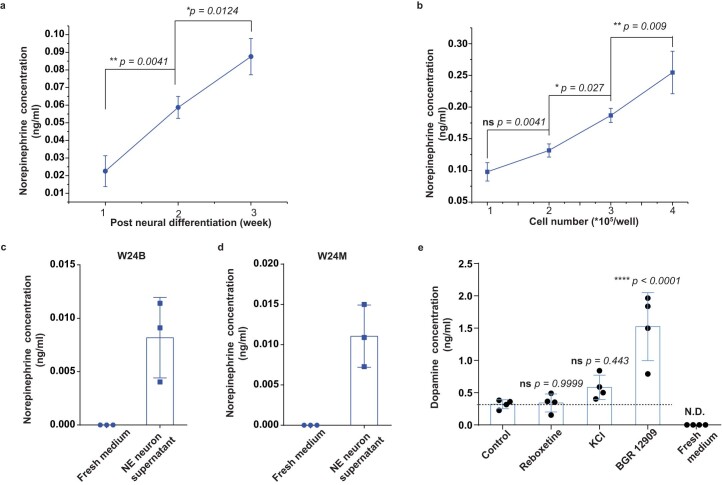
Neurotransmitter release in NE neuron culture. **a–d**, Norepinephrine concentration in the supernate at different time of culture (a), from different density of neuronal cultures (b) and in iPSCs derived NE cells (c,d). Data are shown as mean ± s.d. n = 3 biologically independent samples for each condition. Significance was assessed by one way ANOVA (Sidak’s multiple comparisons test).* p<0.05, ** p <0.01. ns, not significant. **e**, Dopamine. concentration in the supernate collected in different conditions. Data are shown as mean ± s.d. n = 4 biologically independent samples. Significance versus control group was assessed by one way ANOVA (Dunnett’s multiple comparisons test). **** p <0.0001. ns, not significant. N.D., not detected. Source data

ELISA measurement showed that the NE concentration in the culture supernatant increased from 1 week to 3 weeks after plating (Extended Data Fig. 6a), suggesting that NE production correlates with neuronal maturation. At 3 weeks after plating, the NE concentration increased with an increasing number of neurons in each culture (Extended Data Fig. 6b). As controls, fresh media and the supernatant from hPSC-derived cortical neuron cultures for the same period had undetectable NE (Fig. 4j). Furthermore, KCl (40 µM), which depolarizes neurons, significantly increased the NE content (Fig. 4j). Hence, the cultured NE neurons produce and release NE, and its release is activity dependent. NE was also detected in the supernatant from iPSC-derived NE neurons (Extended Data Fig. 6c,d).

The extracellular NE content is regulated by NE re-uptake in vivo. Treatment with NE uptake inhibitors tomoxetine (FDA-approved drug to treat ADHD, depression and anxiety), reboxetine and nisoxetine, but not maprotiline, increased the NE content in the supernatants significantly, whereas the dopamine (DA) and serotonin re-uptake inhibitors (BGR 12909 and citalopram, respectively) did not change the NE content (Fig. 4j), suggesting that NE content is regulated specifically by its re-uptake process in hPSC-derived NE neurons. A small amount of DA was detected in our NE cultures, and its content was increased by DA uptake inhibitor BGR 12909 but not by NE uptake inhibitor reboxetine (Extended Data Fig. 6e). Together, the results indicate that the release and uptake of NE in the hPSC-derived neurons resemble their in vivo counterparts.

## Verification of NE differentiation trajectory by single-nucleus RNA sequencing

**Fig. 5:**
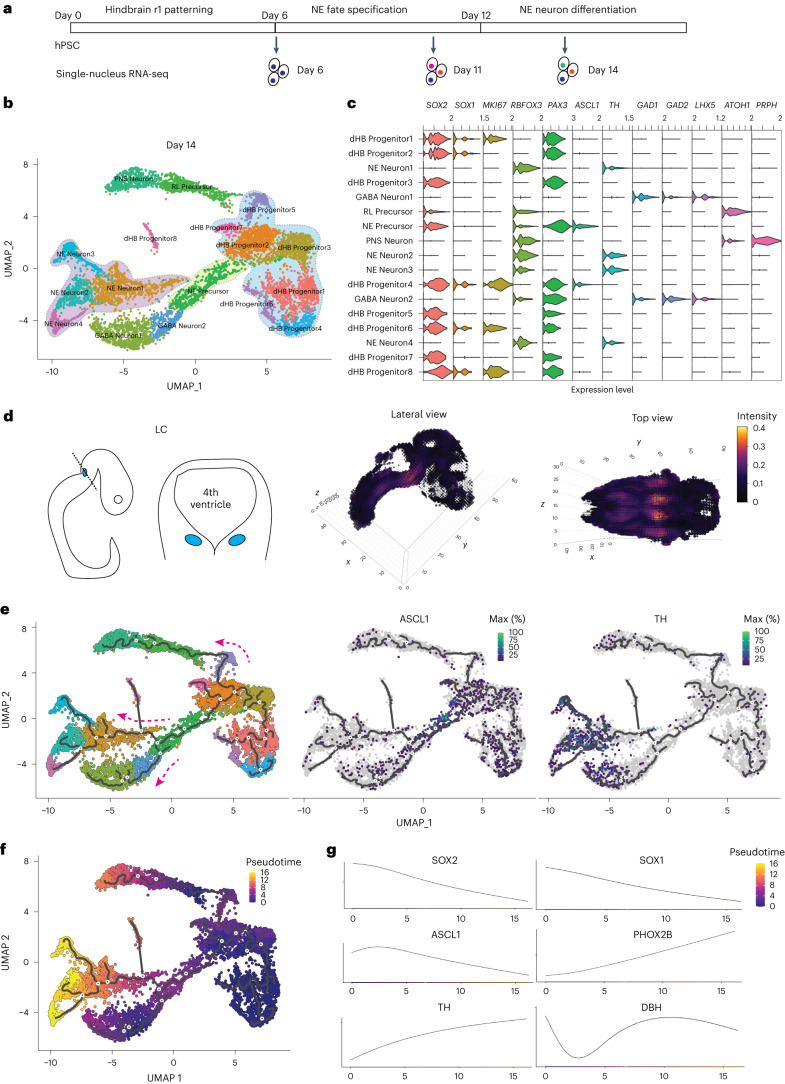
snRNA-seq analysis of the differentiating cells. **a**, Schematic overview of the experimental design. Differentiating cells were collected at day 6, day 11 and day 14 for snRNA-seq. **b**, UMAP embeddings showing clustering of 7,260 cells at day 14 of NE differentiation. Cell clusters were labeled with the cell type annotations. Dorsal hindbrain progenitors, NE precursor and NE neuron clusters are highlighted in the UMAP. **c**, Violin plots of indicated gene expression in all the clusters in **b**. The selected genes were used to annotate the clusters. **d**, Schematic representation of LC location in the mouse neural tube at E10.5 stage of development and the mapping of snRNA-seq cluster (NE Neuron1 in **b**) to the E10.5 Allen Developing Mouse Brain Atlas. **e**, The Monocle trajectory analysis of snRNA-seq data in **b**. The line indicates the differentiation trajectory for the clusters. Three major branches were identified. For NE neurons, the trajectory starts from the dorsal hindbrain progenitors and goes through NE precursors expressing *ASCL1*. **f**, Psedoutime analysis of the snRNA-seq data in **b**. **g**, Dynamic gene expression of neural progenitor and NE markers along the psedoutime.

**Extended Data Fig. 7:**
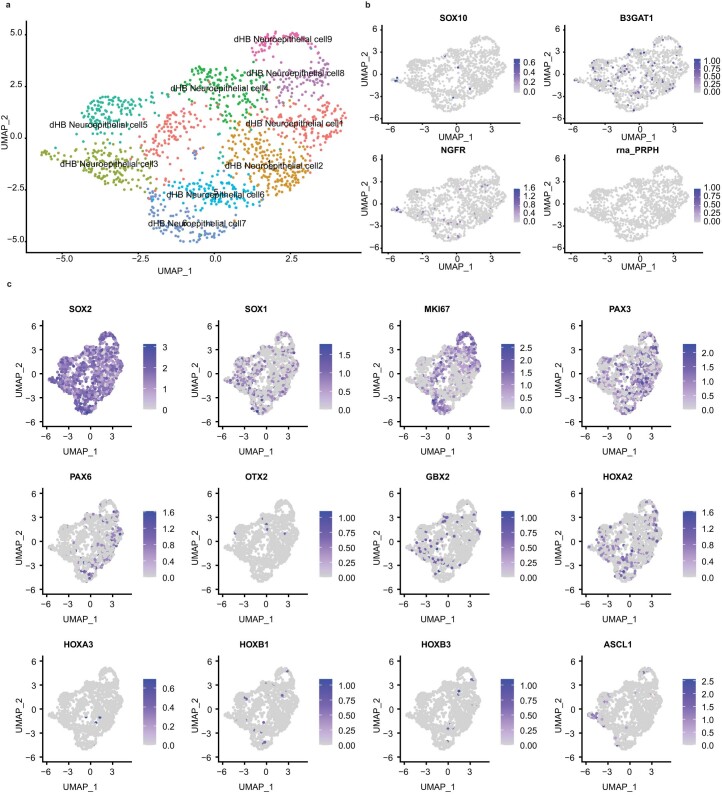
snRNA-Seq analysis of cells under NE differentiation at day 6. **a**, Uniform manifold approximation and projection (UMAP) embeddings showing clustering of differentiating cells at day 6 of NE differentiation. Cell clusters were labeled with cell type annotations. dHB, dorsal hindbrain. **b**, Representative feature plots of genes involved in neural crest stem cell differentiation in the differentiating cells. **c**, Representative feature plots of genes related to brain regions and NE differentiation in the clusters.

**Extended Data Fig. 8:**
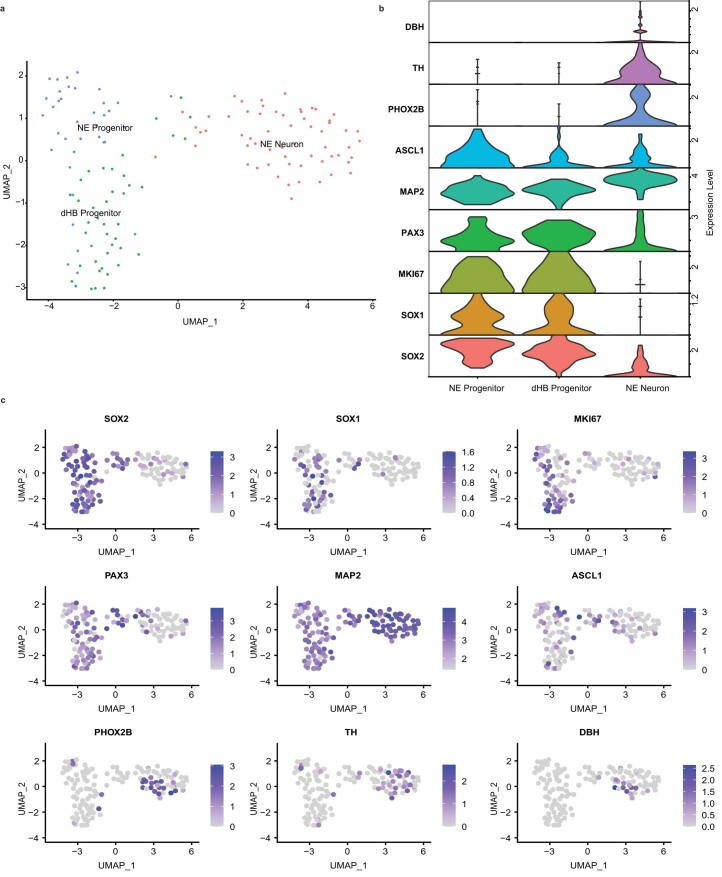
snRNA-Seq analysis of cells under NE differentiation at day 11. **a**, Uniform manifold approximation and projection (UMAP) embeddings showing clustering of differentiating cells at day 11 of NE differentiation. Cell clusters were labeled with cell type annotations. dHB, dorsal hindbrain. NE, Norepinephrine. **b**, Violin plots of gene expression of clusters in a. **c**, Representative feature plots of gene expression in the differentiating cells at day11.

To further study human NE differentiation in vitro, we performed single-nucleus RNA sequencing (snRNA-seq) analysis along the differentiation process. We collected differentiating cells at day 6 (R1 progenitors), day 11 (NE precursors) and day 14 (post-mitotic NE neurons) to capture the key stages of NE differentiation (Fig. 5a). A total of 8,732 high-quality cells were retained for analysis after quality control using the Seurat pipeline^32^. At day 6, nine different cell clusters were identified by uniform manifold approximation and projection (UMAP) clustering (Extended Data Fig. 7a and Supplementary Datasheet 1). There were very few cells expressing neural crest stem cell markers (*SOX10*, *NGFR* and B3GAT1 (also known as *HNK1*)) (Extended Data Fig. 7b). Most of the cells were neuroepithelial cells expressing *SOX2*, *SOX1*, *PAX6* and *PAX3*, with very few cells positive for the forebrain/midbrain marker *OTX2* and caudal hindbrain markers (*HOXA3*, *HOXB1* and *HOXB3*). *GBX2* and *HOXA2* were expressed in those cells at day 6, indicating their rostral hindbrain identity. Consistent with the immunostaining (Supplementary Fig. 1a), there were very few cells expressing the early NE precursor marker *ASCL1* at this stage. At day 11, three major clusters were identified after NE specification (Extended Data Fig. 8a and Supplementary Datasheet 2). Twenty-seven percent of the total cells were in the dorsal hindbrain (dHB) progenitor cluster co-expressing *PAX3*, *SOX2*, *SOX1* and *MKI67*, whereas the early NE progenitor cluster (30%) expressing *ASCL1* linked the dHB progenitors with NE neuron cluster (43%) expressing *PHOX2B*, *TH* and *DBH* (Extended Data Fig. 8b,c). At day 14, the differentiating cells had more diverse cell populations, with 17 clusters identified (Fig. 5b and Supplementary Datasheet 3). Eight clusters were dHB progenitors expressing *SOX2* and *PAX3* (Fig. 5c). Seven clusters were neuronal cells expressing *RBFOX3* (also known as *NEUN*), of which four clusters were NE neurons (22% in all cells, 57% in all neurons) expressing *TH*; two clusters were GABA neurons expressing *GAD1* and *GAD2* and one cluster expressing *PRPH* (Fig. 5c). The other two clusters were post-mitotic neural precursor cells expressing either *ATOH1* (rhombic lip marker) or *ASCL1*. The *ATOH1*-expressing domain is located in a more dorsal part of the *ASCL1* domain in the hindbrain region (Fig. 1e). The cells expressing *PRPH* (called peripheral nervous system (PNS) neurons) were closely linked to the cluster expressing *ATOH1* (called rhombic lip (RL) precursor), suggesting that these neurons may originate from the most dorsal RL cells. Both NE neurons and GABAergic neurons were closely linked with the cluster expressing *ASCL1*, suggesting that both cell types may come from the same progenitor pool. Indeed, hindbrain *ASCL1* progenitors have been shown to give rise to multiple cell types in the brainstem^33^. We also checked mature NE markers, such as *MAOA*, *MAOB*, *DDC*, *COMT*, *ADRA2A/B/C* and *GAL*, in day 14 samples (Supplementary Fig. 4a,b). We found that *MAOA/B* were mainly expressed in the NE Neuron3 cluster, whereas other markers were still sparse in the whole population, indicating the immature status of the newborn neurons at day 14. Indeed, we found that most of the neuronal populations were positive for the immature neuronal marker *DCX*.

**Extended Data Fig. 9:**
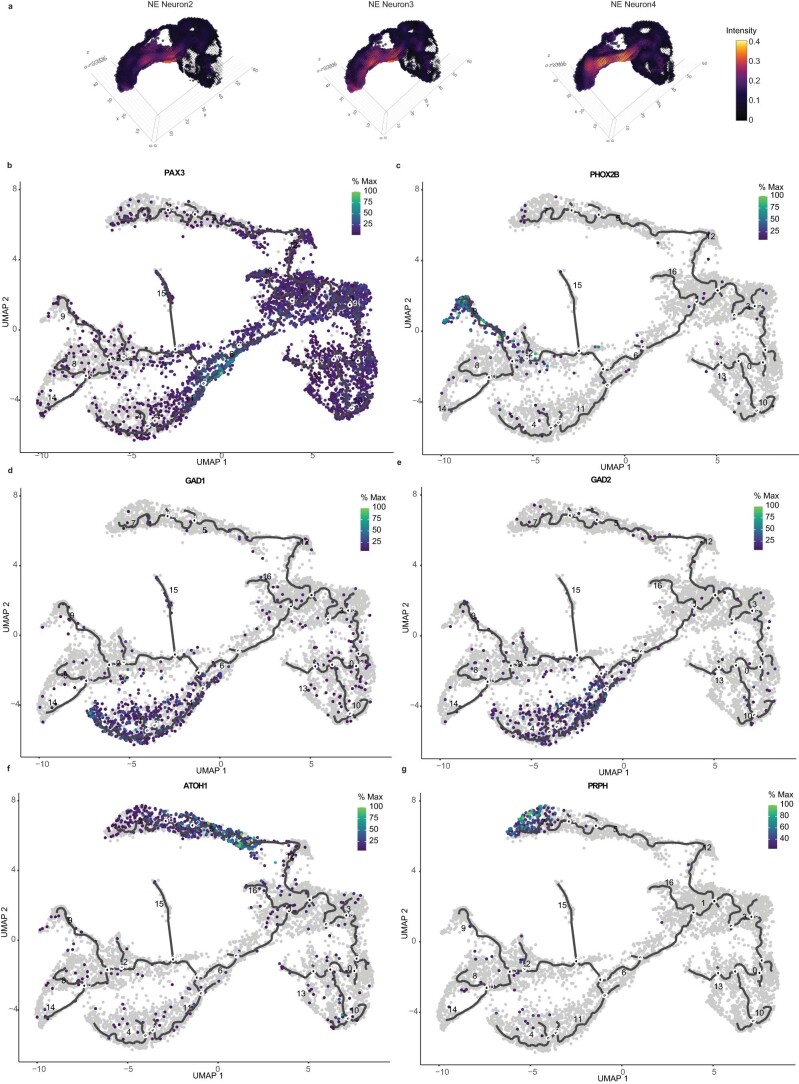
snRNA-Seq analysis of cells under NE differentiation at day 14. **a**, Spatial mapping of NE neural subtypes to the E10.5 Allen Mouse Developing Brain ISH Atlas. **b–g**, Feature plots of selected maker genes in the key trajectory branches towards NE, GABA and peripheral neurons.

We further confirmed the NE identity by VoxHunt^34^ to map the NE neuron clusters to the three-dimensional in situ hybridization (ISH) data from the Allen Developing Mouse Brain Atlas (Fig. 5d). All the NE neuron clusters highly overlayed with the LC region in mouse embryo at embryonic day (E) 10.5 (Fig. 5d and Extended Data Fig. 9a). NE Neuron4 had a broader projection in the mouse embryo than other NE clusters, suggesting that NE subtypes other than the LC-NE may also be present in the culture. Monocle trajectory analysis revealed three major branches in the differentiating cells (Fig. 5e). The dHB progenitor clusters expressing *PAX3* gave rise to three major neuronal cell types (*PRPH*^*+*^, GABA and NE neurons). The trajectory analysis indicates that the NE neurons and GABA neurons were generated through the common precursors expressing *ASCL1*, whereas *PRPH*^+^ PNS neurons were originated from precursors expressing *ATOH1* (Fig. 5e and Extended Data Fig. 9b–g), which is consistent with the known role of *ASCL1* in regulating NE development^21^. Additionally, we performed pseudotime analysis to reveal the dynamic gene expression during the NE differentiation process (Fig. 5f). Neural progenitor markers *SOX2*, *SOX1* and *ASCL1* were gradually downregulated along the pseudotime, whereas NE neuronal markers *PHOX2B*, *TH* and *DBH* were upregulated (Fig. 5g), which is consistent with the qPCR analysis along the NE differentiation (Fig. 4a). Thus, our snRNA-seq analysis confirmed the major NE cell types in the culture and revealed a clear differentiation trajectory of NE neurons from dorsal hindbrain progenitors via *ASCL1*^+^ precursors, as described at the cellular level.

## Chemoreceptor property of LC-NE neurons

**Extended Data Fig. 10:**
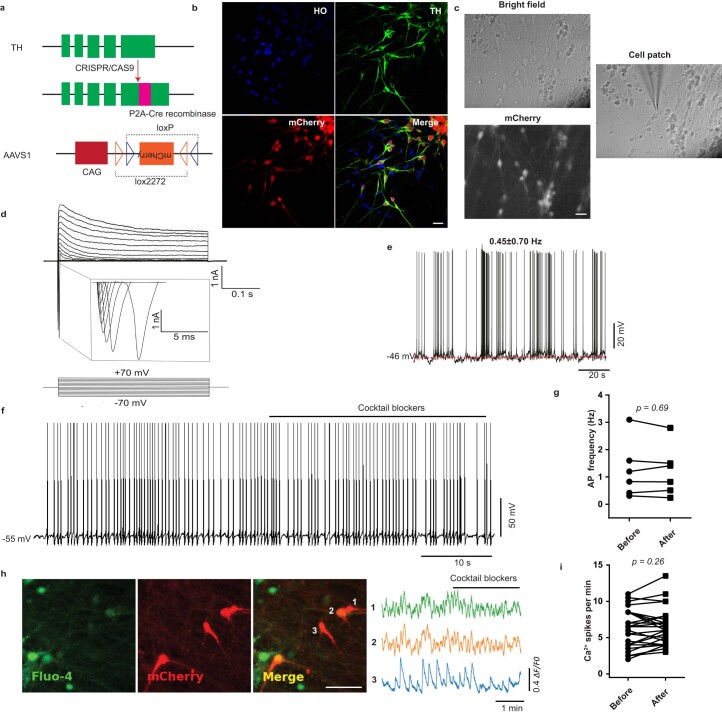
Functional properties of NE neurons. **a**, Schematic diagram of experimental design for generating the TH reporter cell line. **b**, mCherry signal and TH immunostaining in differentiated NE neurons that are differentiated for 7 days. HO, Hoechst. Scale bars, 20 μm. **c**, The bright field and red channel of mCherry fluorescence when performing the cell patch for electrophysiology. Scale bars, 20 μm. **d**, The Na+ and K+ current recorded in NE neurons. **e**, Representative trace of spontaneous firing from recorded NE neurons. **f**, Representative trace of spontaneous firing before, at, and after cocktail blockers. **g**, Quantification of the firing rate change in panel f. Data are shown as symbols and lines in the ‘before-after’ pattern. n = 7 neurons. **h**, Representative image and calcium signals in the TH-labeled neurons before, at, and after cocktail blockers. **i**, Quantification of the firing rate change in panel h. Data are shown as symbols and lines in the ‘before-after’ pattern. n = 26 neurons. Significance was assessed by paired t-test (two tailed) in g, i. Source data

To determine the electrophysiological features of hPSC-derived NE neurons, we built a TH reporter line by inserting the recombinase Cre in the C terminus of the TH gene and a DIO-mCherry element in the AAVS1 site of the genome driven by the CAG promoter (Extended Data Fig. 10a and Supplementary Fig. 5a,b). When the cell expresses TH, the Cre is generated, which results in the mCherry expression. We confirmed the genome editing by DNA sequencing and immunostaining in the TH reporter line, in which mCherry reliably indicated the TH expression (Extended Data Fig. 10b). Under a fluorescence microscope, the labeled NE neurons were readily observed (Extended Data Fig. 10c). Whole-cell patch-clamp recording, performed on 64 neurons at 4–8 weeks after plating (6–10 weeks from hESCs), showed that the mean cell capacitance (Cap) was 25.65 ± 10.28 pF. Inward Na^+^ and outward K^+^ currents were observed in these cells by voltage steps from −70 mV to +70 mV (Extended Data Fig. 10d). About 64.2% (43 of 64) neurons displayed spontaneous action potential (sAP) with an average firing rate at 0.45 ± 0.70 Hz (Extended Data Fig. 10e). Treatment with a cocktail containing antagonists for NMDA receptor (50 µM D-AP5), non-NMDA glutamate receptors (20 µM CNQX), GABA receptor (20 µM bicuculline) and glycine receptor (10 µM strychnine) to block the presynaptic inputs did not significantly change the firing frequency (Extended Data Fig. 10f,g), suggesting the autonomous pacemaker activity in the NE neurons. Along with the sAP firing, we observed large calcium oscillations in the NE neurons using the cell-permeable, fluorescent Ca^2+^ indicator Fluo-4 AM (Extended Data Fig. 10h). Consistent with the pacemaker feature, the calcium oscillation frequency and amplitude were not altered by the cocktail blockers (Extended Data Fig. 10i and Supplementary Fig. 6a). Given the expression of alpha-2 adrenergic receptors in NE neurons (Fig. 4e,i), we asked if NE neurons are regulated by the alpha-2 adrenergic receptor agonist clonidine. Indeed, clonidine (1 mM) reduced the sAP firing rate significantly, which was reversed upon washing away clonidine (Fig. 4l,m).

In rodents, LC-NE neurons increase firing frequency under hypercapnia (high concentration of CO_2_)^35–37^, indicating their chemoreceptor property. We found that about 44% of the recorded NE neurons increased their firing rate when perfused with 5% CO_2_, and the firing frequency went back to baseline once the CO_2_ was washed away (Fig. 4n,o). In contrast, non-TH neurons in the same culture did not increase their firing rate when exposed to 5% CO_2_ (Supplementary Fig. 6b). Thus, the hPSC-derived NE neurons exhibit the characteristic chemoreceptor activity.

## Engineering NE neuron-based sensor cell line for drug screen

**Fig. 6:**
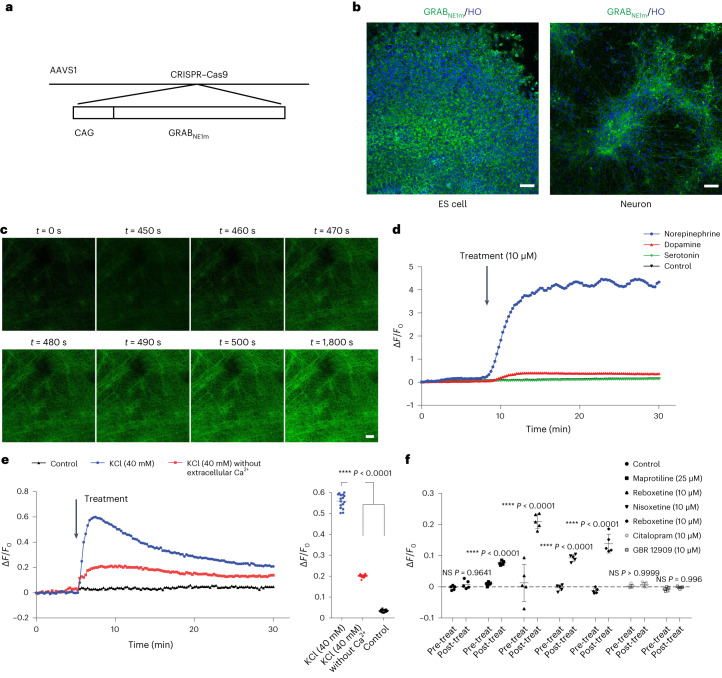
Generation and testing of the NE sensor cells. **a**, Schematic diagram of experimental design for generating the cell line expressing NE sensor GRAB_NE1m_. **b**, GRAB_NE1m_ expression in ESCs and ESC-derived NE neurons. HO, Hoechst. Scale bar, 50 µm. **c**, Time-lapse of GRAB_NE1m_ fluorescence under the treatment of NE. Scale bar, 50 µm. **d**, GRAB_NE1m_ fluorescence intensity along NE (10 µM), DA (10 µM) and serotonin (10 µM) treatment. Δ*F*/*F*_0_ refers to the peak change in fluorescence intensity. The control group overlaps with the serotonin group in the panel. **e**, GRAB_NE1m_ fluorescence intensity along KCl (40 mM) admininstration with or without extracellular calcium. *n* = 16 biologically independent fields for each condition. Significance (versus KCl condition) was assessed by one-way ANOVA (Dunnett’s multiple comparisons test). ****P < 0.0001. **f**, Comparison of fluorescence intensity before and after drug administration. Data are shown as mean ± s.d. *n* = 5 biologically independent samples for both pre-treatment and post-treatment in each condition. Significance was assessed by two-way ANOVA (Sidak’s multiple comparisons test). The comparison is between pre-treatment and post-treatment in each condtion. *P < 0.05, **P < 0.01,***P < 0.001 and ****P < 0.0001. NS, not significant. Source data

To enable testing of drugs that regulate NE release and/or uptake, we engineered, by CRISPR–Cas9, an H9 cell line with a GRAB_NE1m_ sensor^38^ inserted at the AAVS1 site (Fig. 6a and Supplementary Fig. 7a–c), which specifically detects the extracellular NE level. GRAB_NE1m_ sensor, indicated by GFP, was detected in the transgenic ESCs and ESC-derived neurons (Fig. 6b). The fluorescence intensity of the GRAB_NE1m_ sensor increased in response to NE (10 µM) (Fig. 6c,d) but changed little in response to DA (10 µM) and 5-HT (10 µM) (Fig. 6d). The fluorescence intensity changed in response to KCl (40 mM), which was largely attenuated by removing the extracellular calcium (Fig. 6e), suggesting that the GRAB_NE1m_ senses neuronal activity-dependent NE release/uptake.

We then used four NE re-uptake inhibitors (NRIs) (tomoxetine, maprotiline, reboxetine and nisoxetine) to validate the utility of the GRAB_NE1m_ sensor expressing NE neurons for drug testing. All four compounds increased the GRAB_NE1m_ fluorescence intensity significantly (Fig. 6f), whereas the DA and serotonin re-uptake inhibitors, which do not change the extracellular NE concentration (Fig. 4j), did not alter the fluorescence. Thus, this sensor may be useful to find novel drugs that regulate NE release and/or uptake from NE neurons.

## Discussion

We developed a strategy for differentiation of LC-NE neurons from hPSCs at an efficiency of 40–60%. This is achieved by patterning the developing neuroepithelia to dorsal hindbrain r1 identity and subsequently activating the NE program. In particular, we identified the role of ACTIVIN A in specifying NE fate in a temporal and spatial manner. The hPSC-derived NE neurons resemble their in vivo counterparts, displaying extensive axonal arborization, calcium oscillation and pacemaker activities. They release and uptake NE neurotransmitter in an activity-dependent manner, and they possess chemoreceptor activity in response to CO_2_ stimulation. A genetically engineered NE sensor (GRAB_NE1m_) in the hPSC-derived NE neurons reliably reports extracellular NE levels, demonstrating the utility of these neurons for drug screening.

There are multiple NE neuron subtypes in the hindbrain^9,10^. LC-NE neurons originate from the hindbrain r1 region. Thus, it is important to generate neuroepithelia with dorsal hindbrain r1 identity to avoid producing other NE subtypes arising from r2–r5. Activation of the WNT pathway (for example, by CHIR99021) exerts a dose-dependent effect on neuroepithelial patterning along the anterior to posterior axis^39^. This strategy has been used to generate midbrain dopaminergic neurons^40–42^ and hindbrain serotoninergic neurons^20^. Here, fine-tuning of CHIR99021 concentration resulted in efficient generation of hindbrain r1 progenitors. A similar strategy could be used to generate other NE subtypes arising from rhombomeres r2–r5.

Acquisition of regional identity is critical, but often not sufficient, to generate the desired neurons. Studies from model animals indicate that BMPs and FGF8 are essential in acquiring NE fate for the dorsal r1 progenitors^24,43^. However, FGF8 and multiple BMPs have no positive effects on NE fate specification in human cells. In fact, we found that BMPs appear to inhibit NE fate in human progenitors, similar to a previous observation^17^. ACTIVIN A triggers NE neuronal differentiation only in r1 progenitors, but not those in the forebrain, midbrain and more caudal hindbrain regions, suggesting a cell-type-specific effect. Furthermore, ACTIVIN A promotes the transition from ASCL1^+^ NE progenitors to PHOX2A/2B-expressing NE precursors in a dose-dependent manner, again suggesting the specific function of ACTIVIN A in regulating the NE program. Interaction between ACTIVIN A and BMP inhibition (by DMH1) further consolidates the NE fate of the dorsal r1 progenitors. This effect appears independent of regional patterning, as dorsalization by SHH inhibition at this stage has no obvious effect. Both ACTIVIN A and DMH1 act through SMAD signaling^44^, which could provide new avenues for generation of LC-NE neurons.

Forced expression of the NE transcription factor PHOX2A or PHOX2B generates NE neurons from mESCs but with much less efficiency from hPSCs^17^. One possible explanation is that transcription-factor-induced cell fate acquisition coordinates with the molecular machinery in a particular progenitor—in other words, PHOX2A or PHOX2B expression is needed in dorsal r1 progenitors, but not midbrain or more caudal hindbrain progenitors, to confer NE fate. Indeed, we observed that ACTIVIN A turns on the expression of PHOX2A and PHOX2B only in dorsal r1 progenitors.

Chemoreceptor activity is a characteristic activity of LC-NE neurons^45–47^. Chemoreceptor activity and the direct connection of LC-NE neurons with respiratory groups in the brainstem implicate their roles in pathophysiology development in neurological breathing diseases, including congenital central hypoventilation syndrome (CCHS)^6^, sleep disorders^48,49^ and Rett syndrome^50^. However, the neurobiological mechanisms underlying the breathing disorders are unclear. The hPSC-derived NE neurons generated here possess the characteristic functional feature of their counterparts in vivo and will likely be a useful tool to study the cellular and molecular basis by which NE regulates breathing. In addition, LC-NE neurons are dysregulated in many psychiatric conditions^8,12–14^, including ADHD, anxiety and depression, and are often one of the earliest cell types that undergo degeneration in AD and PD. However, little is known about their role in the pathogenesis of these diseases. Thus, human LC-NE neurons may be useful for studying the pathogenesis as well as identifying and testing therapeutics for neurological disorders, as indicated by our LC-NE neurons engineered with an NE sensor.

## Methods

### hPSC culture

hESCs (H9), iPSCs (W24B and W24M) and genetically modified cell lines derived from H9 were maintained on irradiated mouse embryonic fibroblasts (MEFs)^51^. Cells were cultured in the media containing DMEM/F12 basal medium (Thermo Fisher Scientific, 11330-032), 20% KnockOut Serum Replacement (Thermo Fisher Scientific, 10828028), 0.1 mM β-mercaptoethanol, 1 mM l-glutamine (Thermo Fisher Scientific, 25030081), non-essential amino acids (Thermo Fisher Scientific, 11140050) and 4 ng ml^−1^ FGF-2 (Wicell). The cells were passaged weekly by 1 mg ml^−1^ Dispase (Gibco, 17105-041).

### NE neuron differentiation

Two to three days after hPSC passaging, the cells were cultured in the neural induction media consisting of DMEM/F12 (1:1), 1% N2 and 1× non-essential amino acids supplemented with BMP receptor inhibitor DMH1 (2 µM, Tocris, 4126), TGFβ receptor inhibitor SB431542 (2 µM, Stemgent, 04-0010-10) and WNT agonist CHIR99021 (1 μM, Tocris, 4953) (day 0 of NE differentiation) for 4 d. On day 4, the cells were lifted by gently blowing with a 1-ml pipette without digestion. Alternatively, the hPSCs were digested with 1 mg ml^−1^ Dispase for 30 s and cultured in the same media for one additional day in suspension. On day 5, the cultures were fed with the same media but without SB431542. On day 6, ACTIVIN A (25 ng ml^−1^, R&D Systems, 338-AC-050) was added to the culture for 3 d. On day 9, the neural spheres were plated on plates pre-coated with Matrigel and cultured in the neural differentiation media containing DMEM/F12/Neuralbasal (1:1), 1% N2, 2% B27 and 1× non-essential amino acids supplemented with 125 ng ml^−1^ (up to 200 ng ml^−1^) ACITIVIN A and 1 μM c-AMP for another 2–3 d. At around day 10–11, the cells were committed to the NE fate and ready to generate NE neurons.

For NE neuron differentiation, on day 10–11, the cultures were digested into small clusters or single cells by Accutase and cultured in the neural maturation media consisting of neurobasal, 1× B27, 1× non-essential amino acids, 1% GlutaMAX (Gibco, 35050-079) supplemented with 1 μM c-AMP (Sigma-Aldrich, D0627), 0.2 mM ascorbic acid (Tocris, 4055), 10 ng ml^−1^ glial-cell-line-derived neurotrophic factor (GDNF) (PeproTech, 450-10), 10 ng ml^−1^ brain-derived neurotrophic factor (BDNF) (PeproTech, 450-02) and 1 ng ml^−1^ TGFβ1 (PeproTech, 100-21C). Alternatively, NE neuron differentiation was done by changing the culture medium to the neural maturation media without digestion and replating.

### Animals

All animal experiments were conducted according to a protocol approved by the animal care and use committee at the University of Wisconsin-Madison. Adult SCID mice (8–12 weeks) were housed in a pathogen-free environment with a 12-h on and 12-h off day/night cycle, temperature around 24 °C and humidity between 40% and 60%. All animals had access to food and water freely. Cell transplantation was performed as previously described^52^.

### snRNA-seq

The differentiating cells were harvested at indicated timepoints and disassociated by TrypLE (Thermo Fisher Scientific). All cells were collected as pellets by centrifuge after digestion. Samples then underwent nucleus isolation and library construction procedures as described below:

Nucleus isolation: prepare the Lysis Dilution Buffer (10 mM Tris-HCl (pH 7.4; Sigma-Aldrich, T2194), 10 mM NaCl (Sigma-Aldrich, 59222C), 3 mM MgCl_2_ (Sigma-Aldrich, M1028), 1% BSA (Miltenyi Biotec, 130-091-376), 1 mM DTT (Sigma-Aldrich, 646563) and 1 U µl^−1^ RNase inhibitor (Sigma-Aldrich, 3335402001) in nuclease-free water (AmericanBio, AB02123-0500)). Then, 500 µl of chilled 0.1× Lysis Buffer (1× Lysis Buffer: Lysis Dilution Buffer with 0.1% Tween-20 (Bio-Rad, 1662404), 0.1% Nonidet P40 Substitute (Sigma-Aldrich, 74385) and 0.01% digitonin (Thermo Fisher Scientific, BN2006)) was added to the samples to suspend the pellets. The suspension was homogenized in an autoclaved ice-cold 1-ml dounce tissue grinder (DWK Life Sciences, 357538) (30 times with a loose pestle and 30 times with a tight pestle). After a 5-min incubation on ice, the sample was gently pipetted 15 times, followed by a 10-min incubation on ice in 500 µl of Wash Buffer (Lysis Dilution Buffer with 0.1% Tween-20). The solution was filtered through 70-µm (Corning, 352350) and 40-µm (Corning, 352340) tube top cell strainers sequentially (both strainers were pre-wetted with 250 µl of Wash Buffer). The filtered homogenate was then transferred to a 15-ml tube and centrifuged at 500*g* for 5 min at 4 °C using a swing-out rotor (Eppendorf, 5943000343). The pellet was resuspended in 1 ml of Wash Buffer and centrifuged at 500*g* for 5 min at 4 °C. Count the cell number to determine the final resuspension volume after repeating the washing once. The nucleuses were centrifuged at 500*g* for 5 min at 4 °C and then resuspended in the volume calculated in the previous step using Diluted Nuclei Buffer (10x Genomics, 2000153) with 1 mM DTT, RNase inhibitor 1 U µl^−1^ and nuclease-free water. Then, 10 µl of sample was loaded onto a hemocytometer and counted to determine the final concentration.

Library construction: only samples that had a minimum concentration of 3.23 million nuclei per milliliter were used for generation of snRNA-seq libraries using Chromium Next GEM Single Cell Multiome Reagent Kit A (10x Genomics, PN-1000282) following the instructions of the ‘Chromium Next GEM Single Cell Multiome Reagent Kits User Guide’. For snRNA-seq libraries, after pre-amplification, cDNA was constructed, and sample-indexed libraries were generated using Library Construction Kit (10x Genomics, PN-1000190) and Dual Index Kit TT Set A (10x Genomics, PN-1000215) following the manufacturer’s protocol.

### snRNA-seq data analysis

Alignment of raw sequencing reads and generation of feature barcode matrices were done by Cell Ranger (7.1.0). Seurat (4.1.3) was used to process the feature barcode matrices and analyze the snRNA-seq data^32^. All samples were processed under standard quality control. Single cells with more than 600 unique genes (nFeature) and less than 5% mitochondrial gene reads (percent.mt) were selected for follow-up analysis. Additional procedures were used to exclude low-quality cells and doublets in each sample. For day 6 sample, nCount_RNA is set to >4,500, and the total unique gene number is set to <6,000. For day 14 sample, nCount_RNA is set to >2,200, and the unique gene number is set between 1,500 and 5,500. In this study, a total of 8,732 high-quality cells were included in the analysis. After quality control, all the snRNA-seq data were normalized using the SCTransform function in Seurat with the mitochondria genes regressed out. Principal component analysis (PCA), UMAP reduction and gene feature plots were done by using Seurat as well. Trajectory analyses were done by using Monocle3 with the Seurat object^53^. Mapping the snRNA-seq transcriptomic profile to the Allen Developing Mouse Brain ISH Atlas was done by using VoxHunt^34^. All NE neuron clusters at day 14 samples were mapped to the E10.5 mouse embryo ISH atlas (https://developingmouse.brain-map.org/).

### Genome editing

We knocked the Cre recombinase into the TH locus C-terminus and the Grab_NE1m_ into the AAVS1 site by CRSIPR–Cas9 following the published method^54,55^. The sgRNA used for AAVS1 site was from Addgene (plasmid 41818)^56^. The sgRNA (TAGGTGCACGGCGTCCCTGA) for the C-terminus of the TH locus was designed according to Benchling (https://www.benchling.com/). The donor plasmid for the TH reporter was generated by NEBuilder HiFi DNA Assembly Master Mix (New England Biolabs, E2621S). In brief, hPSCs were digested by TrypLE Express. We used Gene Pulser Xcell (Bio-Rad) for CRISPR–Cas9 delivery. Two million cells were electroporated with 15 μg of sgRNA plasmid and 30 μg of donor plasmid. Cells were plated at ∼150,000 cells per well of a six-well plate. Starting from 24 h after electroporation, 0.5 μg ml^−1^ puromycin was added to the culture medium. After 3 d of puromycin selection, hPSCs were switched to their normal culture medium and fed every other day until 1–2 weeks after electroporation when distinct colonies were established. Individual colonies were selected and transferred to 24-well plates (one clone to one well). Then, 3–5 d later, 1–2 clones from each well were picked up to collect the genome DNA for genotyping and DNA sequencing.

### Cell transplantation

All animal experiments were conducted according to a protocol approved by the animal care and use committee at the University of Wisconsin-Madison. In brief, small aggregates of NE neural progenitors (day 10–12) were collected and suspended in artificial cerebral spinal fluid (aCSF) containing Rock inhibitor (0.5 μM), at a concentration of 100,000 cells per microliter. Then, 1 μl of cells was slowly injected into the left cortex (AP = +0.0 mm, ML = +1.8 mm, DV = −1.7 mm, from skull) of adult SCID mice (8–12 weeks) that were anesthetized with 1–2% isoflurane mixed in oxygen. The animals were killed 3 months after transplant for histological analysis, as described^52^.

### Immunocytochemistry and flow cytometry

Immunocytochemistry was performed as described previously^57,58^. In brief, cells on coverslips were fixed in 4% neutral-buffered paraformaldehyde (PFA) for 10 min at room temperature. After rinsing with PBS, they were incubated in 0.2% Triton X-100 (in PBS) for 10 min, followed by 10% donkey serum (in PBS) at room temperature for 1 h. They were then incubated with primary antibodies diluted in 5% donkey serum in 0.1% Triton X-100 (in PBS) at 4 °C overnight, followed by fluorescently conjugated secondary antibodies at room temperature for 30 min. The nuclei were stained with Hoechst. Images were collected with a Nikon A1 laser-scanning confocal microscope.

Flow cytometry was performed using Transcription Factor Buffer Set, which is designed for transcription factor staining, following the manufacturer’s instructions. In brief, single cells were prepared using TrypLE Express Enzyme and fixed in the fixation buffer provided in the kit at 2–8 °C for 45 min. After three washings with the permeable buffer, the primary antibodies were added to cells for 45 min at 2–8 °C in a light-tight box. The cells were washed three times before incubation with fluorescently conjugated secondary antibodies for 45 min at 2–8 °C in a light-tight box. After three times of washing, cells were suspended in washing buffer and analyzed by flow cytometry (BD LSR or BD LSRII). Data analysis was performed using FlowJo (version 10) software.

Primary antibodies used in this study were: OTX2 (1:1,000, AF1979, R&D Systems), EN1 (1:500, 4G11, DSHB), HOAX2 (1:1,000, H9665, Sigma-Aldrich), SOX1 (1:1,000, AF3369, R&D Systems), PAX3/7 (1:200, sc-365843, Santa Cruz Biotechnology), PAX6 (1:1,000, PRB-278P, BioLegend), SOX2 (1:1,000, AF2018, R&D Systems), PHOX2B (1:2,000, AF4940, R&D Systems) or (1:1,000, 66254, Proteintech), ASCL1 (1:500, 556604, BD Biosciences), PHOX2A (1:50, sc-81978, Santa Cruz Biotechnology) or (1:100, ab155084, Abcam), TH (1:1,000, P40101, Pel-Freez Biologicals) and DBH (1:5,000, 22806, Immunostar), neurofilament marker (SMI312) (1:500, 837904, BioLegend), CRHR1 (1:100, 20967-1-AP, Proteintech), orexin receptor 1 (1:500, 18370-1-AP, Proteintech), COMT (1:200, sc-137253, Santa Cruz Biotechnology), NPY (1:1,000, ab30914, Abcam), MOR (1:5,000, 24216, ImmunoStar), ADRA2A (1:100, SAB4500548, MilliporeSigma), PNMT (1:100, AB110, MilliporeSigma), GALANIN (1:500, HPA049864, Sigma-Aldrich), NET (1:1,000, ab211463, Abcam), VGLUT1 (1:500, Synaptic Systems, 135 303), peripherin (1:200, sc-377093, Santa Cruz Biotechnology), CaMKII (1:200, sc-5306, Santa Cruz Biotechnology) and MAO (1:200, sc-271123, Santa Cruz Biotechnology).

Secondary antibodies used in this study were: Alexa Fluor 488 donkey anti-goat IgG (H+L) (1:1,000, A11055, Molecular Probes), Alexa Fluor 546 donkey anti-mouse IgG (1:1,000, A10036, Molecular Probes), Alexa Fluor 488 donkey anti-mouse IgG (H+L) (1:1,000, A21202, Molecular Probes), Alexa Fluor 488, donkey anti-rabbit IgG (H+L) (1:1,000, A21206, Molecular Probes), Alexa Fluor 594 goat anti-rabbit IgG (H+L) (1:1,000, A11037, Molecular Probes) and Alexa Fluor 546 donkey anti-rabbit IgG (H+L) (1:1,000, A10040, Life Technologies).

### ELISA

To detect neurotransmitter release into the media during neuronal differentiation, we collected supernatant at indicated timepoints along differentiation. The cells were treated with drugs (maprotiline 25 µM (Tocris, 0935), tomoxetine 10 µM (Tocris, 2011), nisoxetine 10 µM (Tocris, 1025) and reboxetine 10 µM (Tocris, 1982)) for 4 h or KCl (40 mM) for 30 min before collecting the medium. ELISA was performed by using the Dopamine & Noradrenaline Sensitive ELISA Assay Kit (Eagle Biosciences, BCU39-K02) following the manufacturerʼs instructions.

### Electrophysiology

Whole-cell patch-clamp recordings were done from hESC-derived NE neurons at 4 weeks. In brief, the neurons were held at −70 mV to record the Na^+^/K^+^ channel activities with the voltage-clamp model. For recording action potentials, the cells were held at 0 pA with the current-clamp model and with the steps of injected currents from −50 pA to +50 pA. The bath solution consisted of 135 mM NaCl, 3 mM KCl, 2 mM CaCl_2_, 1 mM MgCl_2_, 10 mM HEPES, 11 mM glucose, 10 mM sucrose, pH 7.4. Hypercapnia condition was produced by bubbling the bath solution with 5% CO_2_ (balanced with oxygen). Recording pipettes were filled with an intracellular solution containing 120 mM potassium d-gluconate, 1 mM ethylene glycol-bis (β-aminoethylether) N,N,N′,N′-tetraacetic acid (EGTA), 10 mM 4-(2-hydroxyethyl)piperazine-1-ethanesulfonic acid (HEPES), 4 mM ATP-Mg, 0.3 mM GTP-Na, 10 mM phosphocreatine, 0.1 mM CaCl_2_, 1 mM MgCl_2_, pH 7.2, 280–290 mOsm L^−1^. An Olympus BX51WI microscope was used to visualize neurons. A MultiClamp 700B amplifer (Axon Instruments, Molecular Devices) was used to investigate the voltage-clamp and current-clamp recordings. Signals were filtered at 4 kHZ using a Digidata 1550B analog/digital converter (Axon Instruments) and stored for further analysis. Data were analyzed with Clampfit 11.0.3 (Axon Instruments), GraphPad Prism 5 (GraphPad Software), CorelDraw 2019 (Corel) and Igor 4.0 (WaveMetrics). Drugs such as clonidine (1 mM) and the cocktail blocker solution containing antagonists for NMDA receptor (50 µM D-AP5, Sigma-Aldrich, A8054), non-NMDA glutamate receptors (20 µM CNQX, Sigma-Aldrich, C239), GABA receptor (20 µM bicuculline, Sigma-Aldrich, 14340) and glycine receptor (10 µM strychnine, Sigma-Aldrich, S8753) were used to treat the cells to examine their effects on NE neuron firing.

### Calcium imaging and analysis

Neuronal Ca^2+^ imaging, image processing and data analysis were performed as described previously with modifications^59^. In brief, cells were bulk-loaded with Fluo-4/AM for 15 min at 37 °C in aCSF containing Fluo-4/AM (12.5 μg ml^−1^), pluronic acid (0.05%) and DMSO (0.1%). Then, cells were transferred to a chamber, and Ca^2+^ imaging was performed with a Nikon A1 confocal microscope at room temperature. All image data were taken in the frame-scanning mode at four frames per second. The Ca^2+^ imaging data were analyzed using Python. Ca^2+^ signals were presented as relative fluorescence changes (Δ*F*/*F*_0_) from specified regions of interest (ROIs). In this experiment, only mCherry^+^ cells were selected for analysis. For the traces with baseline drift, baseline correction was performed using a rolling ball algorithm. The peaks were detected using the algorithm developed by MATLAB (findpeaks function). The frequency and amplitude were calculated and measured. Images with obvious motion were excluded for analysis. In experiments that examined spontaneous Ca^2+^ oscillations, the Ca^2+^ level was reported as Δ*F*/*F*_0_ = (*F*_t_ − *F*_0_) / *F*_0_. Calcium elevation events were detected with thresholds of three times of s.d. of the baseline.

### Fluorescence imaging of GRAB_NE1m_ sensor cells

The neurons were plated following the NE differentiation protocol and cultured on a 35-mm glass-bottom dish (well size 14 mm, #1.5 glass-like polymer coverslip (D35-14-1.5P, Cellvis)) until day 30 for maturation. Expression GRAB_NE1m_ in hPSC-derived NE neurons was imaged live under the Nikon A1 confocal microscope. The cells were allowed to condition at room temperature after changed to the electrophysiological buffer consisting of 135 mM NaCl, 3 mM KCl, 2 mM CaCl_2_, 1 mM MgCl_2_, 10 mM HEPES, 11 mM glucose, 10 mM sucrose, pH 7.4. Then, the cells were recorded for 6–7 min under ×20 objective with PFS on to monitor the dynamic fluorescence intensity change before treatments with neurotransmitters (NE, DA, 5-HT or control solution) or drugs (DA, 5-HT and NRIs) at indicated concentration. The extracellular NE level changes were quantified by Δ*F*/*F*_0_ = (*F*_t_ − *F*_0_) / *F*_0_ using ImageJ (1.53q).

### Statistics and reproducibility

Statistical analyses were performed using GraphPad Prism 5 or Microsoft Excel software. The methods used to assess the significance are specified in the figure legends. The exact statistical values are provided in figures and the source data used for plotting in the respective figures. Representative data, such as qPCR and immunostaining shown in all the figures, were repeated at least three times independently with similar results.

### Reporting summary

Further information on research design is available in the Nature Portfolio Reporting Summary linked to this article.

## Online content

Any methods, additional references, Nature Portfolio reporting summaries, source data, extended data, supplementary information, acknowledgements, peer review information; details of author contributions and competing interests; and statements of data and code availability are available at 10.1038/s41587-023-01977-4.

## Supplementary information

Supplementary Information Supplementary Figs. 1–7.

Reporting Summary

Supplementary Table **Supplementary Table 1** Cluster markers for day 6 snRNA-seq. **Supplementary Table 2** Cluster markers for day 11 snRNA-seq. **Supplementary Table 3** Cluster markers for day 14 snRNA-seq.

Supplementary Data 1 Statistical source data and statistical test result for Supplementary Fig. 1.

Supplementary Data 2 Statistical source data and statistical test result for Supplementary Fig. 6.

## Source data

Source Data Fig. 1 Statistical source data and statistical test result.

Source Data Fig. 2 Statistical source data and statistical test result.

Source Data Fig. 3 Statistical source data and statistical test result.

Source Data Fig. 4 Statistical source data and statistical test result.

Source Data Fig. 6 Statistical source data and statistical test result.

Source Data Extended Data Fig. 1 Statistical source data and statistical test result.

Source Data Extended Data Fig. 2 Statistical source data and statistical test result.

Source Data Extended Data Fig. 3 Statistical source data and statistical test result.

Source Data Extended Data Fig. 4 Statistical source data and statistical test result.

Source Data Extended Data Fig. 6 Statistical source data and statistical test result.

Source Data Extended Data Fig. 10 Statistical source data and statistical test result.

## Data Availability

The raw snRNA-seq datasets are available at the Gene Expression Omnibus with accession number GSE221988 (datasets are GSM6911289, GSM6911290 and GSM6911291). Allen Developing Mouse Brain Atlas is from the Allen Brain Atlas Data Portal (http://help.brain-map.org/display/devmouse/API). All other raw data used for plotting in the figures are provided as Source Data; statistical tests and results are provided in the Source Data as well. Source data are provided with this paper.
