# Supplementary material for: Generation of locus coeruleus norepinephrine neurons from human pluripotent stem cells
Source: Nat Biotechnol. 2023 Nov 16;42(9):1404–16. doi: 10.1038/s41587-023-01977-4 (PMC11392812; doi:10.1038/s41587-023-01977-4)
Supplement: Supplementary file 1 — Supplementary Figs. 1–7. [file 41587_2023_1977_MOESM1_ESM.pdf]

nature biotechnology

Article

https://doi.org/10.1038/s41587-023-01977-4

# Generation of locus coeruleus norepinephrine neurons from human pluripotent stem cells

In the format provided by the authors and unedited

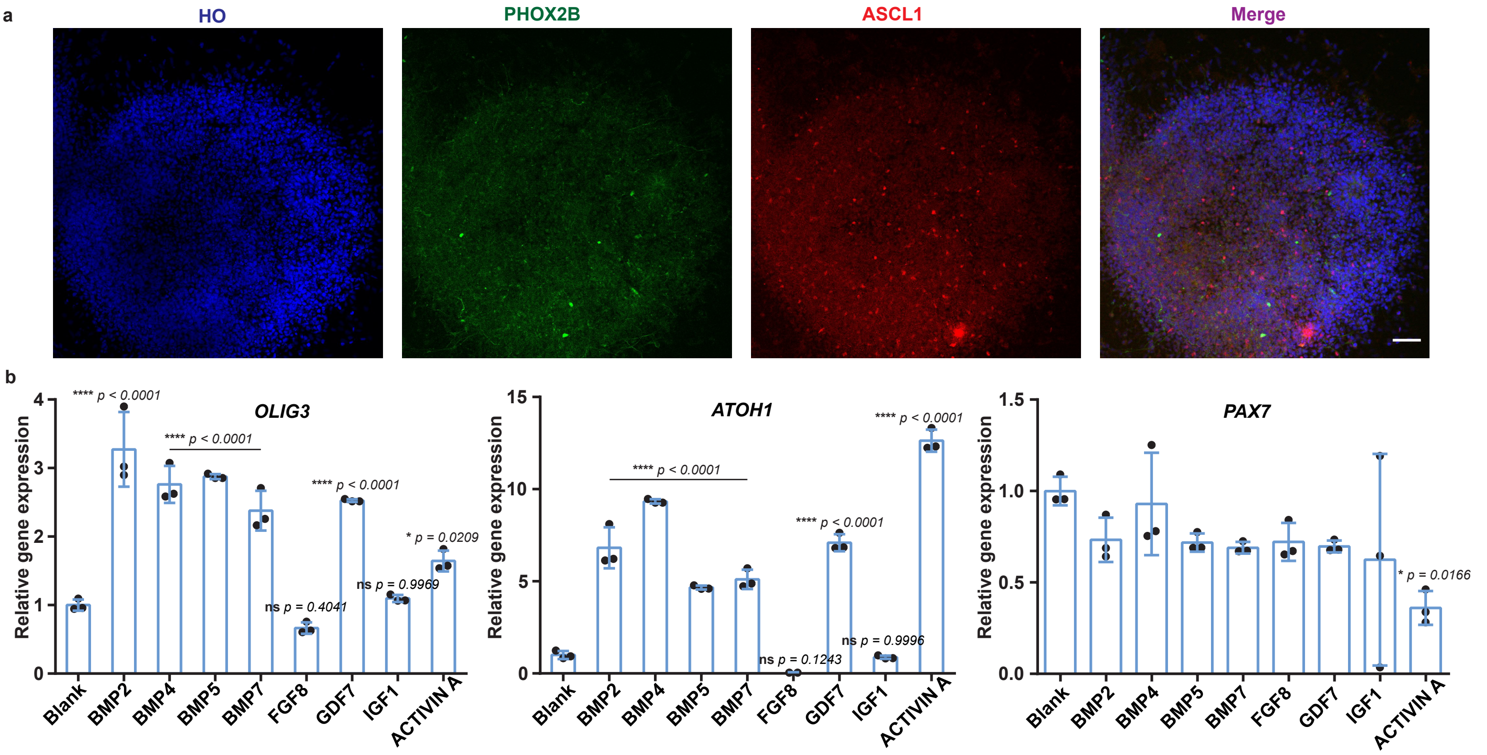


**Supplementary Figure 1 NE fate specification a,**Immunostaining for norepinephrine neural progenitor markers ASCL1 and PHOX2B at day 6 from cells treated with 1.0 μM CHIR99021. HO, Hoechst. Scale bar, 50 μm. **b,** qPCR of regional neural progenitor markers *OLIG3*, *ATOH1* and *PAX7* under the treatment of BMPs, FGF8,GDF7, IGF1 and ACTIVIN A. Data are shown as mean ± SD. n = 3 biologically independent samples. The significance compared to “Blank” condition was assessed by one way ANOVA (Dunnett’s comparisons test). * p<0.05, ** p <0.01,*** p <0.001 and ****p <0.0001. ns, not significant.

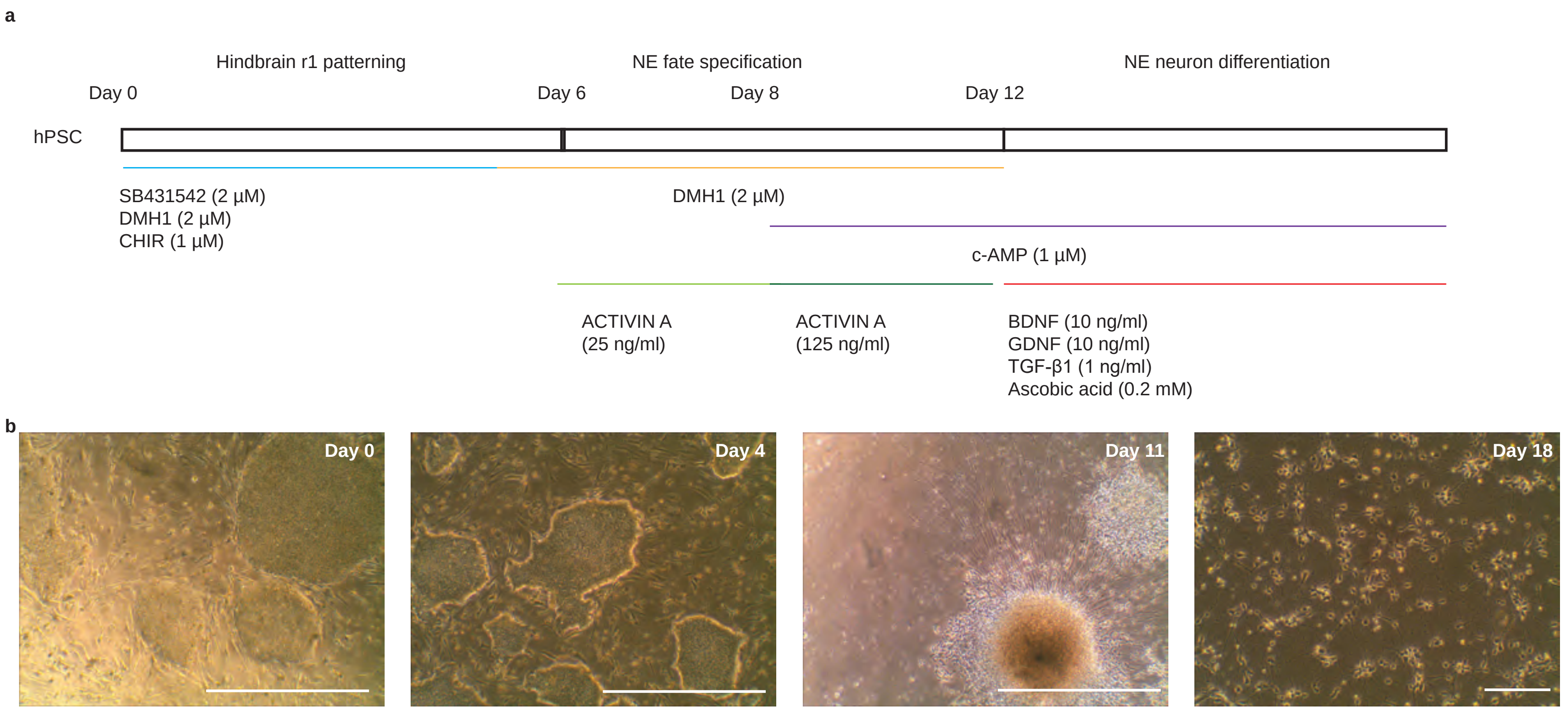


**Supplementary Figure 2 NE neuron differentiation a,** The diagram of the NE neural differentiation protocol. **b,** The bright field view of cells at different time points along NE neural differentiation. Scale bars, 1 mm.

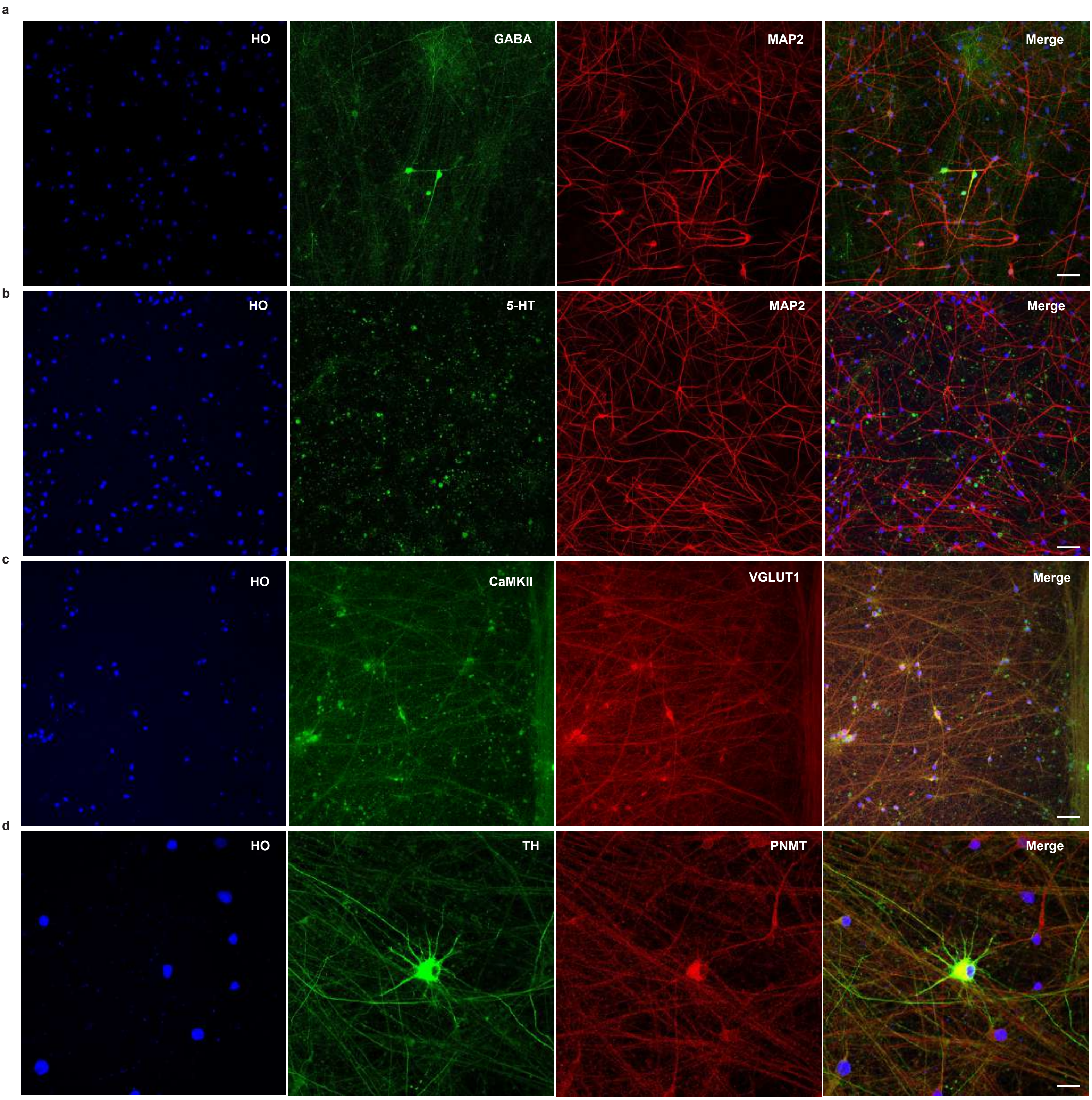


**Supplementary Figure 3 NE neuron differentiation (a-d),** immunostaining for GABA, 5HT VGLUT1, CaMKII and PNMT in NE neurons at day 30. HO, Hoechst. Scale bars in (a-c), 50 μm; Scale bar in (d) , 20 μm;.

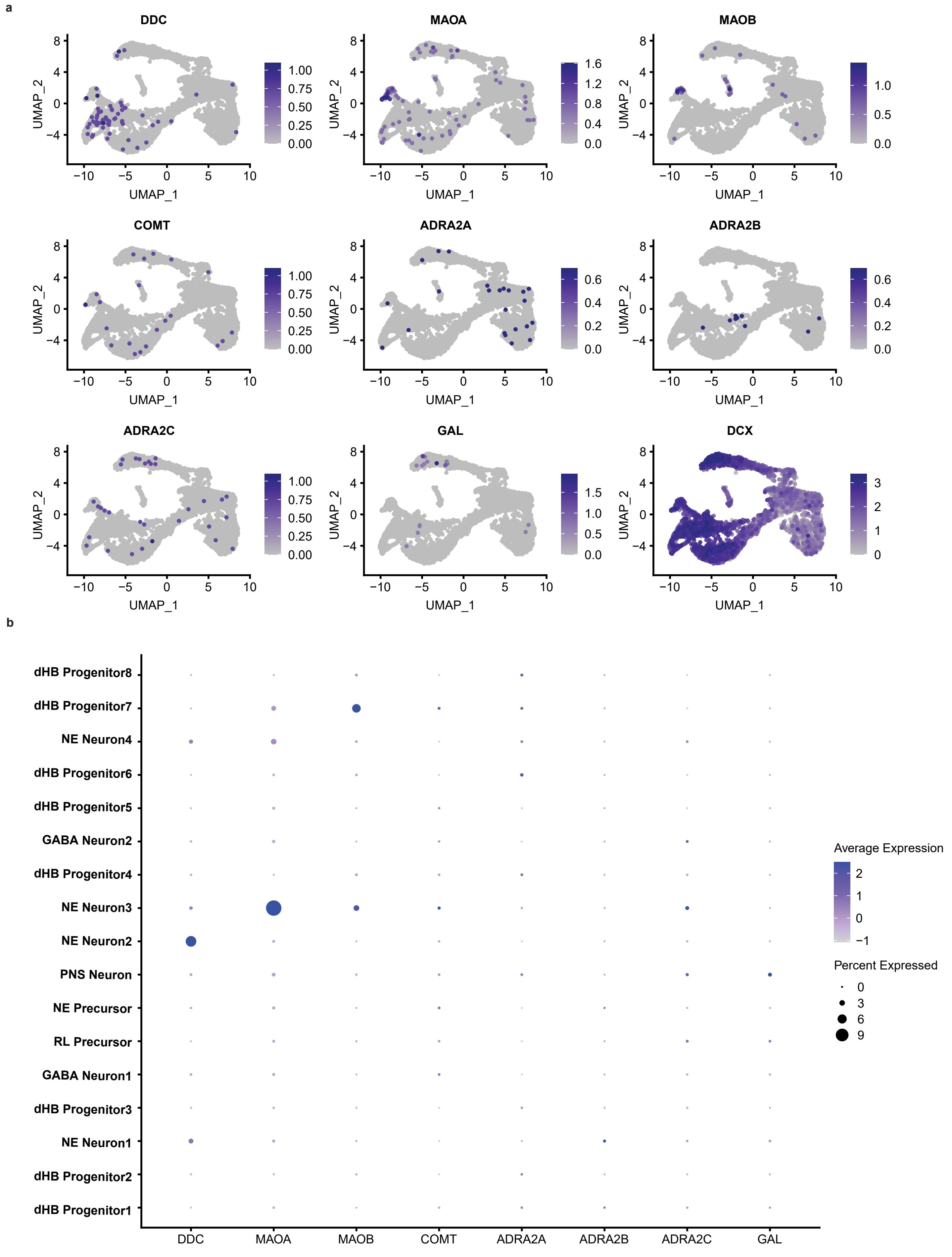


**Supplementary Figure 4 NE neuronal marker expression at day 14 a,** Feature plots of later NE markers in day 14 sample. **b,** Dot plots of later NE markers in each cluster of day 14 snRNA-Seq dataset.

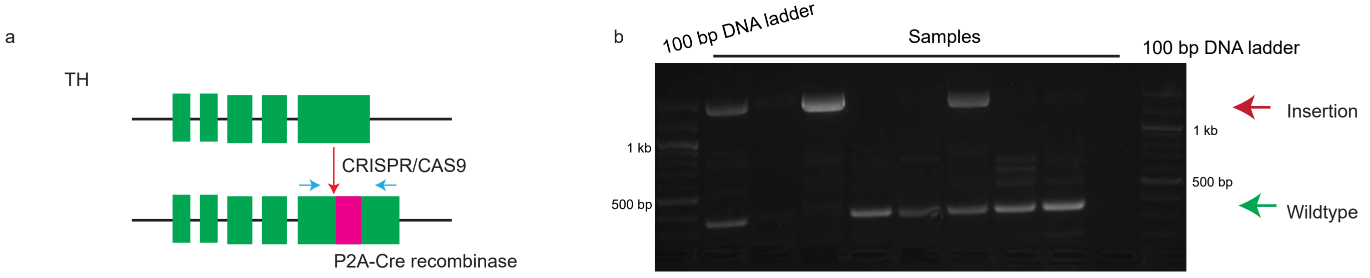


**Supplementary Figure 5 Generation of TH reporter cell line a,** Schematic diagram of experimental design for generating the TH reporter cell line. The blue arrows indicate the primer designed for genotyping. **b,** The genotyping PCR of the candidate cell lines. The red arrow and green arrow indicate the insertion and wildtype bands respectively.

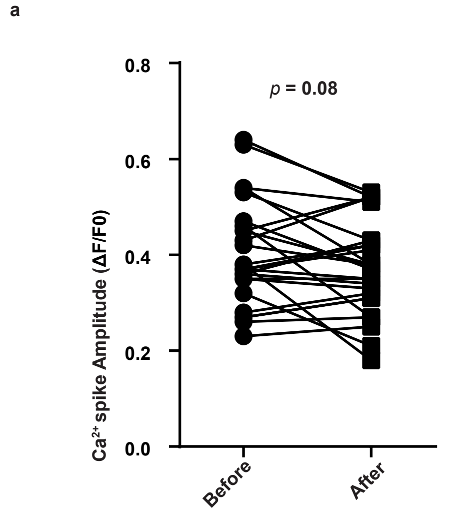


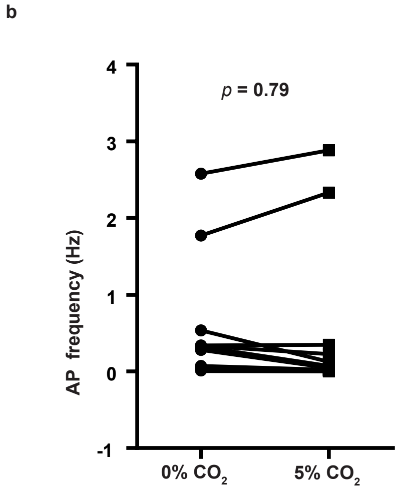


**Supplementary Figure 6 Functional properties of NE neurons a,** Quantification of calcium spike amplitude in NE neurons before, at, and after cocktail blockers. Data are shown as symbols and lines in the “before-after” pattern. n = 26 neurons. Significance was assessed by paired t-test (two tailed).
**b,** Quantification of firing rate change in non-TH cells from the NE differentiating culture. Data are shown as symbols and lines in the “before-after” pattern. n = 14 neurons. Significance was assessed by paired t-test (two tailed).

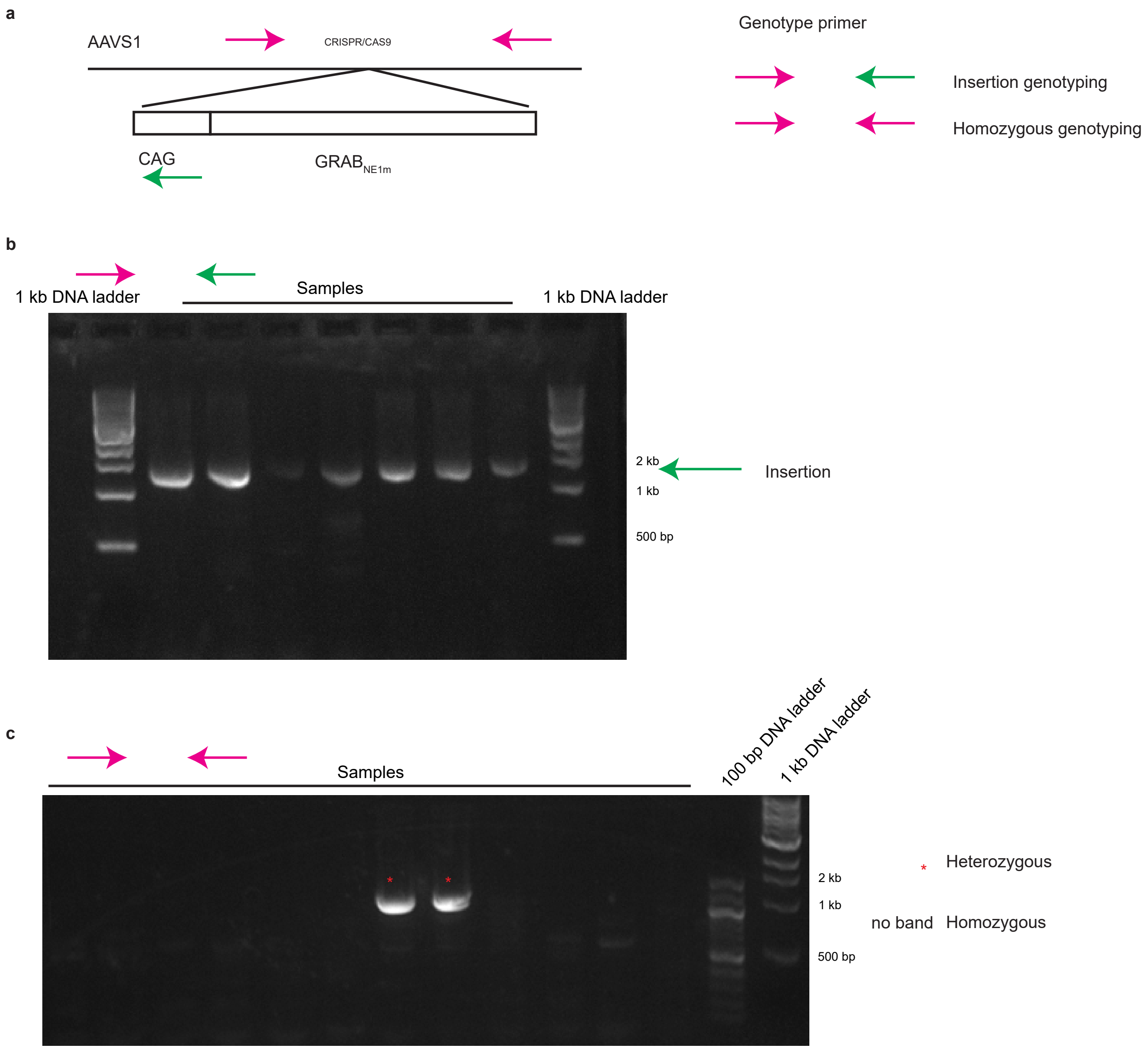


**Supplementary Figure 7 Generation of NE sensor cell line a,** Schematic diagram of experimental design for generating cell line expressing norepinephrine sensor GRAB$_{NE1m}$. The genotyping primers were shown by the arrows. **b,c,** The genotyping PCR of the candidate cell lines using primers to detect insertion (**b**) and homozygousity (**c**).
