# Supplementary material for: Generation of locus coeruleus norepinephrine neurons from human pluripotent stem cells
Source: Nat Biotechnol. 2023 Nov 16;42(9):1404–16. doi: 10.1038/s41587-023-01977-4 (PMC11392812; doi:10.1038/s41587-023-01977-4)
Supplement: Supplementary file 2 — Reporting Summary [file 41587_2023_1977_MOESM2_ESM.pdf]

nature research

Corresponding author(s): Su-chun Zhang

Last updated by author(s): Aug 25, 2023

Reporting Summary

Nature Research wishes to improve the reproducibility of the work that we publish. This form provides structure for consistency and transparency in reporting. For further information on Nature Research policies, see our Editorial Policies and the Editorial Policy Checklist.

Statistics

For all statistical analyses, confirm that the following items are present in the figure legend, table legend, main text, or Methods section.

n/a	Confirmed	
☐	☒	The exact sample size (n) for each experimental group/condition, given as a discrete number and unit of measurement
☐	☒	A statement on whether measurements were taken from distinct samples or whether the same sample was measured repeatedly
☐	☒	The statistical test(s) used AND whether they are one- or two-sided<br>*Only common tests should be described solely by name; describe more complex techniques in the Methods section.*
☐	☒	A description of all covariates tested
☒	☐	A description of any assumptions or corrections, such as tests of normality and adjustment for multiple comparisons
☐	☒	A full description of the statistical parameters including central tendency (e.g. means) or other basic estimates (e.g. regression coefficient) AND variation (e.g. standard deviation) or associated estimates of uncertainty (e.g. confidence intervals)
☐	☒	For null hypothesis testing, the test statistic (e.g. F, t, r) with confidence intervals, effect sizes, degrees of freedom and P value noted<br>*Give P values as exact values whenever suitable.*
☒	☐	For Bayesian analysis, information on the choice of priors and Markov chain Monte Carlo settings
☒	☐	For hierarchical and complex designs, identification of the appropriate level for tests and full reporting of outcomes
☒	☐	Estimates of effect sizes (e.g. Cohen's d, Pearson's r), indicating how they were calculated

Our web collection on statistics for biologists contains articles on many of the points above.

Software and code

Policy information about availability of computer code

Data collection	qPCR data was collected by CFX manager software (version 3.1, BIORAD). Flow cytometry data was collected by the FACS software installed on a BD LSR Fortessa.
Data analysis	The following softwares have been used for data analysis: Cellranger (7.1.0), Seurat (4.1.3), ImagJ (1.53q), FlowJo (V10), GraphPad Prism 5 , Excel (office365),

For manuscripts utilizing custom algorithms or software that are central to the research but not yet described in published literature, software must be made available to editors and reviewers. We strongly encourage code deposition in a community repository (e.g. GitHub). See the Nature Research guidelines for submitting code & software for further information.

Data

Policy information about availability of data

All manuscripts must include a data availability statement. This statement should provide the following information, where applicable:

- Accession codes, unique identifiers, or web links for publicly available datasets
- A list of figures that have associated raw data
- A description of any restrictions on data availability

The raw snRNA-Seq datasets are available at GEO with accession number GSE221988 (datasets are GSM6911289, GSM6911290 and GSM6911291). Allen Developing Mouse Brain Atlas is from ALLEN BRAIN ATLAS DATA PORTAL (http://help.brain-map.org/display/devmouse/API). All other raw data used for plotting in the figures are provided as source data and statistical tests and results were provided in the source data as well.

Field-specific reporting

Please select the one below that is the best fit for your research. If you are not sure, read the appropriate sections before making your selection.

☒ Life sciences ☐ Behavioural & social sciences ☐ Ecological, evolutionary & environmental sciences

For a reference copy of the document with all sections, see nature.com/documents/nr-reporting-summary-flat.pdf

Life sciences study design

All studies must disclose on these points even when the disclosure is negative.

Sample size	at least n = 3 were used for each condition. The sample size were determined by previous study (Tao et al., Nature Medicine 2021)
Data exclusions	no data was excluded
Replication	two additional hiPSC were used to verify the described protocol. The representative data such as qPCR and immunostaining shown in Figures have been repeated at least 3 times independently with similar results. for each batch, 3 biological replicates were used for experiments.
Randomization	The sample allocation is random throughout the study.
Blinding	The quantification of immunostaining data is blinding. The person quantifying the images didn't what samples are those and which groups they belong.

Reporting for specific materials, systems and methods

We require information from authors about some types of materials, experimental systems and methods used in many studies. Here, indicate whether each material, system or method listed is relevant to your study. If you are not sure if a list item applies to your research, read the appropriate section before selecting a response.

Materials & experimental systems

n/a	Involved in the study
☐	☒ Antibodies
☐	☒ Eukaryotic cell lines
☒	☐ Palaeontology and archaeology
☐	☒ Animals and other organisms
☒	☐ Human research participants
☒	☐ Clinical data
☒	☐ Dual use research of concern

Methods

n/a	Involved in the study
☒	☐ ChIP-seq
☐	☒ Flow cytometry
☒	☐ MRI-based neuroimaging

Antibodies

Antibodies used	Primary antibodies used in this study were: OTX2 (1:1000, AF1979, R&D systems), EN1 (1:500, 4G11, DSHB), HOAX2 (1:1000, H9665, Sigma), SOX1 (1:1000, AF3369, R&D systems), PAX3/7 (1:200, sc-365843, Santa Cruz Biotechnology), PAX6 (1:1000, PRB-278P, Biolegend), SOX2 (1:1000, AF2018, R&D systems), PHOX2B (1:2000, AF4940, R&D systems) or (1:1000, 66254, Proteintech), ASCL1 (1:500, 556604, BD Biosciences), PHOX2A (1:50, sc-81978, Santa Cruz Biotechnology) or (1:100, ab155084, Abcam), TH (1:1000, P40101, Pel-Freez Biologicals) and DBH (1:5000, 22806, Immunostar), Neurofilament Marker (SMI312) (1:500, 837904, BioLegend), CRHR1 (1:100, 20967-1-AP, Proteintech), Orexin receptor 1 (1:500, 18370-1-AP, Proteintech), COMT (1:200, sc-137253, Santa Cruz Biotechnology), NPY (1:1000, ab30914, abcam), MOR (1:5000, 24216, Immunostar), ADRA2A (1:100, SAB4500548, Millipore Sigma), PNMT (1:100, AB110, Millipore Sigma), GALANIN (1:500, HPA049864, Sigma-Aldrich), NET (1:1000, ab211463, abcam) , VGLUT1 (1:500, Synaptic System, 135 303), Peripherin (1:200, sc-377093 , Sant Cruz Biotechnology), CaMKII (1:200, sc-5306, Santa Cruz Biotechnology) and MAO (1:200, sc-271123, Santa Cruz Biotechnology). Secondary antibodies used in this study were: Alexa Fluor 488 donkey anti-goat IgG (H+L) (1:1000, A11055, Molecular Probes), Alexa Fluor 546 Donkey Anti-Mouse IgG (1:1000, A10036, Molecular Probes), Alexa Fluor 488 Donkey Aanti-Mouse IgG (H+L) (1:1000, A21202, Molecular Probes), Alexa Fluor 488, Donkey anti-Rabbit IgG (H+L) (1:1000, A21206, Molecular Probes), Alexa Fluor 594 goat anti-rabbit IgG (H+L) (1:1000, A11037, Molecular Probes) and Alexa Fluor 546 donkey anti-rabbit IgG (H+L) (1:1000, A10040, Life Technologies).
Validation	OTX2 (1:1000, AF1979, R&D systems) has been validated in 81 publications and is routinely used in our lab to target human OTX2, EN1 (1:500, 4G11, DSHB) has been validated in 12 publications and is routinely used in our lab to target human EN1, HOAX2 (1:1000, H9665, Sigma) has been validated by the producer in human samples, SOX1 (1:1000, AF3369, R&D systems) has been validated in more than 60 publications and is routinely used in our lab to target human SOX1; PAX3/7 (1:200, sc-365843, Santa Cruz Biotechnology) has been used by 7 publications targeting both human and mouse PAX3/7. we also see clear labeling with this antibody; PAX6 (1:1000, PRB-278P, Biolegend) has been used in more than 300 publications and is routinely used in our lab to target human PAX6; SOX2 (1:1000, AF2018, R&D systems) has been used and validated by 195 publications and is routinely used in our lab; PHOX2B (1:2000, AF4940, R&D systems) has been validated by the producer and 3 publications. we observed clear labeling with this

antibody; or (1:1000, 66254, Proteintech) is validated by co-staining with the other PHOX2B antibody. we observed complete overlay between these two; ASCL1 (1:500, 556604, BD Biosciences) has been widely used in the research and is routinely used in our lab, PHOX2A (1:50, sc-81978, Santa Cruz Biotechnology) has been validated by the producer; or (1:100, ab155084, Abcam) has been validated by the producer; TH (1:1000, P40101, Pel-Freez Biologicals) has been validated by the producer and numerous publications. we used it routinely; DBH (1:5000, 22806, Immunostar) has been validated by the company and this antibody received good comments from buyers; Neurofilament Marker (SMI312) (1:500, 837904, BioLegend) has been tested in human neurons with specific labeling; CRHR1 (1:100, 20967-1-AP, Proteintech), Orexin receptor 1 (1:500, 18370-1-AP, Proteintech), COMT (1:200, sc-137253, Santa Cruz Biotechnology), NPY (1:1000, ab30914, abcam), MOR (1:5000, 24216, Immunostar), ADRA2A (1:100, SAB4500548, Millipore Sigma), PNMT (1:100, AB110, Millipore Sigma), GALANIN (1:500, HPA049864, Sigma-Aldrich), NET (1:1000, ab211463, abcam) , VGLUT1 (1:500, Synaptic System, 135 303), Peripherin (1:200, sc-377093 , Sant Cruz Biotechnology), CaMKII (1:200, sc-5306, Santa Cruz Biotechnology) and MAO (1:200, sc-271123, Santa Cruz Biotechnology) have been validated by the producer and many publications. PRPH and GAL has been validated using mouse brain tissue in this study. Secondary antibodies used in this study were: Alexa Fluor 488 donkey anti-goat IgG (H+L) (1:1000, A11055, Molecular Probes), Alexa Fluor 546 Donkey Anti-Mouse IgG (1:1000, A10036, Molecular Probes), Alexa Fluor 488 Donkey Aanti-Mouse IgG (H+L) (1:1000, A21202, Molecular Probes), Alexa Fluor 488, Donkey anti-Rabbit IgG (H+L) (1:1000, A21206, Molecular Probes), Alexa Fluor 594 goat anti-rabbit IgG (H+L) (1:1000, A11037, Molecular Probes) and Alexa Fluor 546 donkey anti-rabbit IgG (H+L) (1:1000, A10040, Life Technologies) are routinely used in the lab with high quality.

Eukaryotic cell lines

Policy information about cell lines

Cell line source(s)	H9 were purchased from Wicell. W24B and W24M iPSC were from Dr. Anita Bhattacharyya lab
Authentication	H9 were authenticated by STR profiling at Wicell. W24B and W24M were anthenticated by STR profiling by Anita Bhattacharyya lab.
Mycoplasma contamination	All cell lines were tested negatively for Mycoplasma
Commonly misidentified lines (See ICLAC register)	No misidentified lines used in this study

Flow Cytometry

Plots

Confirm that:

- [x] The axis labels state the marker and fluorochrome used (e.g. CD4-FITC).
- [x] The axis scales are clearly visible. Include numbers along axes only for bottom left plot of group (a 'group' is an analysis of identical markers).
- [x] All plots are contour plots with outliers or pseudocolor plots.
- [x] A numerical value for number of cells or percentage (with statistics) is provided.

Methodology

Sample preparation	Flow cytometry was performed using Transcription Factor Buffer Set which is designed for transcription factor staining following manufacturer's instruction. Briefly, single cells were prepared using TrypLE Express Enzyme and fixed in the fixation buffer provided by the kit at 2-8°C for 45 min. After 3 washings with the permeable buffer, the primary antibodies were added to cells for 45 min at 2-8°C in a light-tight box. The cells were washed 3 times before incubation with fluorescently conjugated secondary antibodies for 45 mins at 2-8°C in a light-tight box. After 3 times of washing, cells were suspended in washing buffer and analyzed by flow cytometry
Instrument	BD LSR or BD LSRII
Software	FlowJo
Cell population abundance	10000 events were recorded for each sample
Gating strategy	unstained negative control were used as gate reference

- [ ] Tick this box to confirm that a figure exemplifying the gating strategy is provided in the Supplementary Information.
